# Persistent high latitude amplification of the Pacific Ocean over the past 10 million years

**DOI:** 10.1038/s41467-022-35011-z

**Published:** 2022-11-27

**Authors:** Xiaoqing Liu, Matthew Huber, Gavin L. Foster, Andrew Dessler, Yi Ge Zhang

**Affiliations:** 1 Department of Oceanography, Texas A&M University, College Station, TX 77843 USA; 2 Department of Earth, Atmospheric and Planetary Sciences, Purdue University, West Lafayette, IN 47907 USA; 3 School of Ocean and Earth Science, University of Southampton, National Oceanography Centre Southampton, Southampton, SO14 3ZH UK; 4 Department of Atmospheric Sciences, Texas A&M University, College Station, TX 77843 USA

**Keywords:** Palaeoclimate, Palaeoceanography

## Abstract

While high latitude amplification is seen in modern observations, paleoclimate records, and climate modeling, better constraints on the magnitude and pattern of amplification would provide insights into the mechanisms that drive it, which remain actively debated. Here we present multi-proxy multi-site paleotemperature records over the last 10 million years from the Western Pacific Warm Pool (WPWP) – the warmest endmember of the global ocean that is uniquely important in the global radiative feedback change. These sea surface temperature records, based on lipid biomarkers and seawater Mg/Ca-adjusted foraminiferal Mg/Ca, unequivocally show warmer WPWP in the past, and a secular cooling over the last 10 million years. Compiling these data with existing records reveals a persistent, nearly stationary, extratropical response pattern in the Pacific in which high latitude (~50°N) temperatures increase by ~2.4° for each degree of WPWP warming. This relative warming pattern is also evident in model outputs of millennium-long climate simulations with quadrupling atmospheric CO_2_, therefore providing a strong constraint on the future equilibrium response of the Earth System.

## Abstract (web summary)

Multiproxy SST reconstructions of the Western Pacific Warm Pool show cooling over the entire last 10 Ma. High latitude Pacific Ocean SSTs are shown to be amplified, warming 2.4 °C per 1° of warming in the WPWP. This is reproduced by climate models.

## Introduction

When the Earth warms, high latitudes often warm more than low latitudes, a phenomenon commonly known as “high latitude amplification”. The past, present, and future of high latitude amplification have been studied extensively using climate models and paleoclimate data, but the pattern and magnitude of amplification remain uncertain and are areas of great concern due to the impacts of future warming on sea ice decline, atmospheric circulation changes and extreme weather, and carbon release associated with permafrost melting^1–4^. In particular, debate continues with regard to the relative roles of albedo, water vapor, and lapse rate feedbacks in determining high latitude amplification, with the pattern of warming itself potentially affecting the sensitivity of the climate system to radiative forcing due to changes in greenhouse gas concentrations^5,6^. Exploring time intervals with and without significant polar ice may provide key insights into elucidating these different feedbacks^4,7,8^. Investigating high latitude amplification over the past 10 million years (Myr) of Earth’s history when the Northern Hemisphere evolved from a largely ice-free condition to one with major continental glaciers and sea ice, therefore, provides invaluable insights into the causes and expressions of high latitude amplification^7,9^. Of particular interest is how the pattern of warming behaves on different timescales and how that informs our understanding of future warming patterns with implications for global temperatures^10^.

Quantitative sea surface temperature (SST) reconstructions provide a basis for establishing temperature gradients and for determining high latitude amplification in the geological past^8^. To reconstruct SSTs, the algal biomarker-based alkenone unsaturation index ${{{{{{\rm{U}}}}}}}_{37}^{{{{{{\rm{K}}}}}}^{\prime} }$ is a well-validated, accurate proxy^11^. It has been widely used to establish middle to high-latitude SST records, with existing records covering most of the late Miocene (since ~12 million years ago, Ma) to the present^12,13^. However, the ${{{{{{\rm{U}}}}}}}_{37}^{{{{{{\rm{K}}}}}}^{\prime} }$ index reaches its maximum value of 1 at about 29 °C^14^, limiting its application in warm regions, especially the Western Pacific Warm Pool (WPWP) defined by the 28.5 °C isotherms in the modern ocean. Being the warmest and largest surface water body, the WPWP dominates global oceanic heat transport^15^ and also drives major atmospheric circulations such as the latitudinal Hadley Cell and longitudinal Walker Cell^16^. Historical data and climate simulations show that the ascent area of the WPWP exerts the dominant control on the global radiative feedback^17^.

**Fig. 1:**
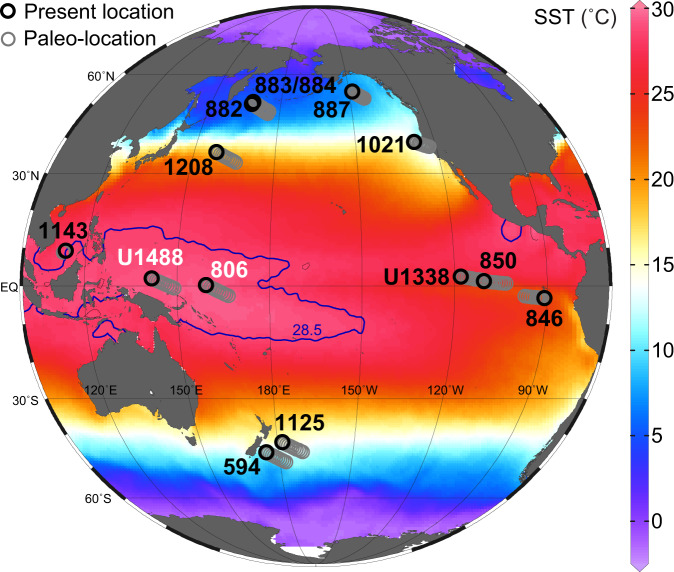
Pacific sites used in this study. Sites with raw data from this study are labeled in white: International Ocean Discovery Program (IODP) Site U1488 and Ocean Drilling Program (ODP) Site 806. Black labels indicate sites with data obtained from previous studies: IODP Site U1338, ODP Sites 1143, 850, 846, 1208, 1021, 882, 883/884, 887, 1125, and Deep Sea Drilling Project (DSDP) Site 594. Gray circles represent the paleo-locations of each site at a 1-million-year window and black circles indicate present locations. Blue contour lines represent the 28.5 °C isotherm that defines the modern Western Pacific Warm Pool. The map was generated by Ocean Data View software using the temperature data from World Ocean Atlas 2013 and colors represent the statistical mean of annual sea surface temperature (SST) from 1955 to 2012.

The temperatures of the WPWP are critical since they define the equator-to-pole gradients and high latitude amplification as this region represents the endmember with the warmest SSTs for determining energy budgets^1^. The deficiencies of the ${{{{{{\rm{U}}}}}}}_{37}^{{{{{{\rm{K}}}}}}^{\prime} }$ proxy have required reliance on other proxies, such as the biomarker-based TEX_86_ proxy and the foraminiferal Mg/Ca-based proxy, to determine the evolution of the WPWP, with conflicting results. For instance, when applied to Ocean Drilling Program (ODP) Sites 806 (0°19.1′N, 159°21.7′E) and 1143 (9°21.72′N, 113°17.11′E, Fig. 1), TEX_86_ revealed a long-term cooling^18,19^, in contrast to marine carbonate-based approaches (e.g., Mg/Ca and Δ_47_) that suggested the WPWP was thermally stable since the Pliocene (~5 Ma)^20,21^. This has become a contentious issue, with questions being raised about the veracity of the proxies used, the corrections applied and the sites chosen^22,23^. Thus, ambiguity with regard to the thermal evolution of the WPWP remains and currently limits our understanding of the evolution of Pacific temperature gradients and high latitude amplification in particular.

Here we present multi-proxy (${{{{{{\rm{U}}}}}}}_{37}^{{{{{{\rm{K}}}}}}^{\prime} }$, TEX_86_, Mg/Ca), multi-site (U1488, 806) SST records of the WPWP for the past 10 Myr, improving the spatial coverage within the WPWP and providing a comprehensive view of the evolution of ocean temperature in this important region. These data, together with published SSTs from the extratropics of the Pacific Ocean, are then used to evaluate the pattern of ocean temperature changes relative to the WPWP, the result of which is also compared with climate model outputs.

## Results and discussions

### SST records of the WPWP

The ${{{{{{\rm{U}}}}}}}_{37}^{{{{{{\rm{K}}}}}}^{\prime} }$- and TEX_86_-derived SSTs are based on sediments of Site U1488 recovered by the recent International Ocean Discovery Program (IODP) Expedition 363 which provides unique material to study the evolution of the central part of the WPWP since the Miocene. Samples from Site U1488 (02°02.59′N, 141°45.29′E, Fig. 1), located on the Eauripik Rise north of Papua New Guinea, were subjected to biomarker analyses and SST estimates, supplemented by planktonic foraminiferal Mg/Ca measurements from the late Miocene samples of ODP Site 806 (12.5–5.8 Ma) (Supplementary Table 1). The total organic carbon content of sediments from Site U1488 is overall low, averaged to ~0.2 wt%^24^. We, therefore, extracted lipids from large samples (~20–60 g), which helped to obtain sufficient alkenones and glycerol dialkyl alycerol tetraethers (GDGTs) for reliable ${{{{{{\rm{U}}}}}}}_{37}^{{{{{{\rm{K}}}}}}^{\prime} }$ and TEX_86_ determinations (Supplementary Fig. 1). Site 806 is situated on the Ontong Java plateau (Fig. 1), with abundant planktic foraminifera *Trilobatus sacculifer* present in the late Miocene section which was used to extend the existing Mg/Ca record of the Plio-Pleistocene^20^.

The application of paleothermometers, particularly on older sediments, has to be accompanied by thorough evaluations of the limitations and caveats of each proxy. For example, the distribution of GDGTs in sediment samples could be influenced by non-thermal effects, which would invalidate the application of TEX_86_ for SST reconstructions. We, therefore, screened the GDGT data using a series of criteria including ring index, methane index, branched and isoprenoid tetraether index, GDGT-0/crenarchaeol, %GDGT-0, and %GDGT-2 (see the section “Methods”, Supplementary Fig. 2). For Site U1488, the majority of the data (89%) passed these vigorous tests and therefore was included in our SST estimate. Several recent studies have suggested that on a global scale, the TEX_86_ signal derives from the surface or within the top 200 m of the water column, validating its usage as a surface or shallow subsurface ocean temperature proxy^25,26^. This is especially likely to be true in the WPWP with upper thermocline depths of 100–200 m^27^. At Site U1488, GDGT-2/GDGT-3 ratio (used to detect the GDGTs with a deep-water origin^28,29^) is low overall (<6.1), supporting the utilization of TEX_86_ as an SST proxy. Finally, we used a Bayesian-based spatially varying regression (BAYSPAR) calibration^30^ to convert TEX_86_ to SSTs, including consideration of the small paleogeography changes of the sites.

On multi-million-year timescales, the Mg/Ca paleothermometer is complicated by the potential for carbonate diagenesis and long-term seawater Mg/Ca (Mg/Ca_sw_) variations. The diagenetic impact on trace metals is not fully understood, although nanoscale secondary ion mass spectrometry results suggest that foraminiferal Mg/Ca is relatively robust^31^. However, numerous lines of evidence, such as halite-hosted fluid inclusions^32^, calcium carbonate veins formed on mid-ocean ridge flanks^33^, sediment core pore-fluid profiles^34^, and biogenic carbonates^35^ have shown that for the past 10 Myr, Mg/Ca_sw_ has increased substantially and therefore has to be considered when applying the Mg/Ca thermometry.

**Fig. 2:**
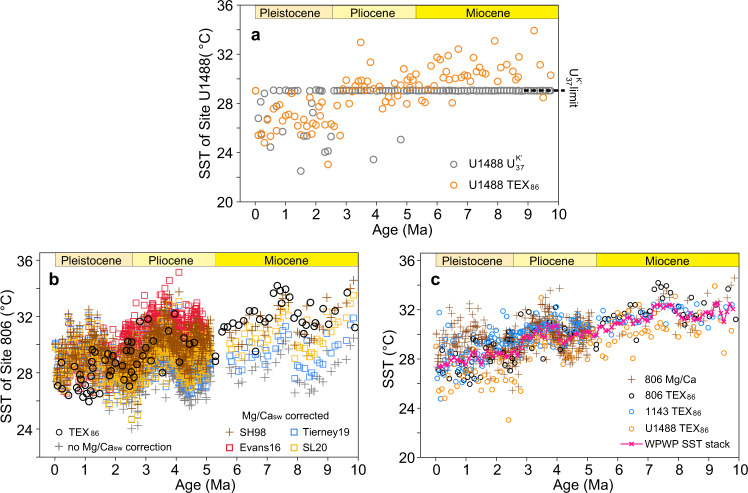
Sea surface temperature reconstructions of the Western Pacific Warm Pool over the past 10 million years. **a** sea surface temperature (SST) of Site U1488 derived from TEX_86_ (orange circles) and ${{{{{{\rm{U}}}}}}}_{37}^{{{{{{\rm{K}}}}}}{\prime} }$ (gray circles) proxies. The dashed line represents the maximum allowable temperature (~29 °C) of the ${{{{{{\rm{U}}}}}}}_{37}^{{{{{{\rm{K}}}}}}{\prime} }$ proxy. **b** Site 806 Mg/Ca-SST with and without Mg/Ca_sw_ correction, in comparison with TEX_86_-SST (black circles) of the same site. Brown crosses and red, blue and yellow squares represent Mg/Ca_sw_ scenarios from Stanley and Hardie^100^ (SH98), Evans et al.^37^(Evans16), Tierney et al.^38^ (Tierney19), and Sosdian and Lear^39^ (SL20), respectively. **c** stacked SST of the Western Pacific Warm Pool (WPWP). This stack (pink line) was calculated with TEX_86_-SST from Sites U1488 (orange circles), 806 (black circles), 1143 (blue circles), and Mg/Ca-SST (brown crosses) from Site 806 based on the Mg/Ca_sw_ of SH98.

Here we applied a geochemical model-derived Mg/Ca_sw_ scenario by Stanley and Hardie^36^ to adjust the influence of varying Mg/Ca_sw_ on the Mg/Ca paleothermometry (Fig. 2b), given that this simulated Mg/Ca_sw_ agree well with the proxy data and is available for the entire studied interval (Supplementary Fig. 3). This numerically modeled result was obtained by accounting for the steady-state mixing of riverine input and mid-ocean ridge hydrothermal brines. Other modeled or proxy-based Mg/Ca_sw_ scenarios (Supplementary Fig. 3), such as back-calculating Mg/Ca_sw_ using biomarker-derived SSTs and planktonic foraminiferal Mg/Ca^37^, and fitting a regression to all proxy data^38^ and to all but fossil coral data^39^, were also used to compute Mg/Ca-derived SSTs (Fig. 2b). These different Mg/Ca_sw_ scenarios yield different SST history of Site 806 (Fig. 2b). Nonetheless, it is critical to point out that any Mg/Ca_sw_ correction of Mg/Ca-derived SSTs results in the decrease of Site 806 temperatures from the late Miocene to the Pliocene and Pleistocene, in stark contrast with the uncorrected SSTs which do not show a clear trend over the last 10 Myr (Fig. 2b).

Data generated during this study and existing SST records from Sites U1488, 806, and 1143^18,19^ in the central, eastern, and western parts of the WPWP provide the unique opportunity to broadly evaluate the temperature changes of the warm pool over the past 10 Myr. The excellent agreement between the independent proxies from the same site (i.e., Site 806, Fig. 2c and Supplementary Fig. 4) and sites that are thousands of kilometers away (Fig. 2c) demonstrates these approaches are individually robust and together, they unambiguously show that the WPWP was warmer during the late Miocene–Pliocene than the present, and document a secular cooling since 10 Ma (Fig. 2c), despite the SST data scatter from ~2 Ma towards the present associated with large glacial-interglacial temperature variations. ${{{{{{\rm{U}}}}}}}_{37}^{{{{{{\rm{K}}}}}}^{\prime} }$-SST from Site U1488 show a range of values for the most recent 3 Myr, but they are mostly pinned to the maximum value between 10 and 3 Ma (Fig. 2a), similar to the previous results from Sites 806 and 1143^18,40^. This qualitatively supports the notion of a warmer than present WPWP during the Mio-Pliocene. These results contradict the “permanent El Niño” theory^20^, which argues that the WPWP was “stable” since the early Pliocene. A “stable” WPWP along with a much warmer cold tongue in the Eastern Equatorial Pacific (EEP) (Supplementary Fig. 5) led to nearly absent zonal temperature gradients across the equatorial Pacific during the Pliocene, resembling modern “El Niño” events. However, the zonal SST gradients calculated using our WPWP SST record were never below 1 °C over the past 10 Myr (Supplementary Fig. 6), unsupportive of a permanent El Niño-like mean climate state, but allowing for time intervals with a smaller gradient than today’s. Also, perhaps with greater importance^5^, our results confirm that the WPWP does respond to global warming during the Pliocene and Miocene^12,13,18^, in agreement with the predictions of theory and climate models with important implications for the future climates^41^.

### Pacific SSTs, SST gradients, and high latitude amplification

**Fig. 3:**
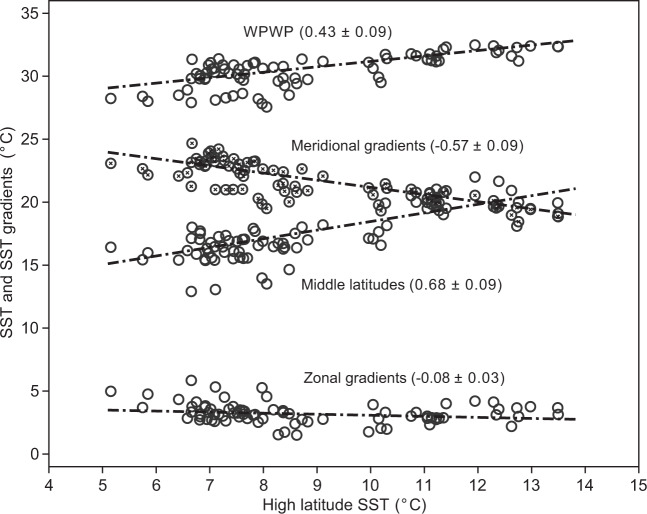
WPWP and middle latitude temperatures and meridional and equatorial zonal temperature gradients plotted against the corresponding high latitude SSTs over the last 10 Myr. Meridional gradients are sea surface temperature (SST) differences between the Western Pacific Warm Pool (WPWP) and the high latitudes, and zonal gradients are SST differences between the WPWP and the Eastern Equatorial Pacific. All dash-dotted lines show simple linear regressions except for meridional “gradients”, which was calculated in a way that avoids the apparent interdependence between meridional gradients and high-latitude SST (see the “Methods” section). The numbers in parentheses represent the slope of the linear relationship with one standard error.

Here we evaluate the meridional temperature gradients and high latitude amplification of the Pacific Ocean with our WPWP temperature estimates and published SST records from the EEP and extra-tropics (Fig. 1, Supplementary notes 1, 2, Supplementary Table 2 and Supplementary Figs. 5, 7 and 8). This was achieved through a novel approach that normalizes the magnitude from paleoclimate data to a metric that enables *direct comparison with modern climate and climate modeling without having to consider the changes in the temporal domain*. The procedure is as follows:

1. Stack SSTs from the WPWP, middle latitudes (30°−50°) and high latitudes (>50°) separately using a time-binning approach, to yield regional-averaged SST changes (Supplementary Fig. 9, see the section “Methods”). This treatment shows that similar to the WPWP, middle latitude and high latitude SSTs also exhibit an overall decline since 10 Ma (Supplementary Figs. 9, 10), but with transient deviations from this secular trend (Supplementary Figs. 8, 9).
2. Use these stacked data to calculate high latitude amplification over the past 10 Myr. To do this, we ordinated the SST data in all regions by high latitude SST (Fig. 3), rather than time as is customary. This allows us to focus on how temperatures elsewhere vary as a function of high latitude SSTs, with the understanding that high latitudes are responding to a variety of possible forcings such as greenhouse gas, icesheet, and volcanic forcing and thus those are implicitly represented in these formulations of the SST data from other regions ordinated by high latitude SST. Specifically, the WPWP and mid-latitude temperatures and temperature differences from the WPWP to high latitudes are compared directly against high latitude temperatures (Fig. 3), since the SST data were binned for every 200 thousand years (kyr) and then stacked for the WPWP, mid-latitude, and high-latitude regions (Supplementary Fig. 9).

High latitude amplification is clearly expressed by the decrease in meridional SST gradients (WPWP—high latitude) with increasing high latitude temperatures (Fig. 3). This is consistent with observations of the more distant Mesozoic and early Cenozoic greenhouse climates which are characterized by reduced meridional gradients^42^. The high latitude amplification factor can be further quantified by a weighted linear regression (York Regression^43^) between the WPWP and high latitude temperatures over the last 10 Myr (Fig. 3), which is equivalent to 1/slope of the linear regression. It should be noted that our definition of Pacific “high latitude amplification” is different from the more commonly used “polar” or “Arctic amplification”, which cannot be constrained here since polar SSTs with the timespan and resolution equivalent to our Pacific records are currently unavailable.

**Fig. 4:**
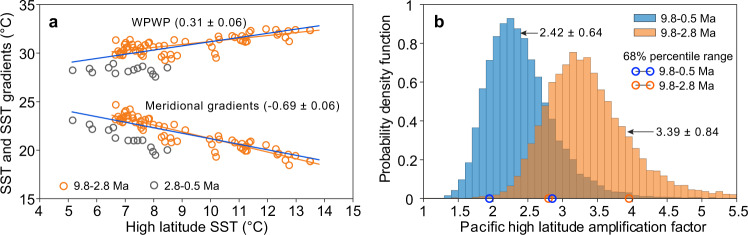
Pacific high latitude amplification of the ice-free (10–2.8 Ma) Northern Hemisphere vs. the past 10 Myr. **a** Sea surface temperature (SST) changes of the Western Pacific Warm Pool (WPWP) relative to high latitudes. Meridional gradients are SST differences from the WPWP to high latitudes. Orange dots represent the data prior to 2.8 Ma, whereas gray dots represent the data after 2.8 Ma. Orange lines indicate linear regressions for the data prior to 2.8 Ma. The regression methods are the same as those in Fig. 3 and Table 1. Blue lines represent linear regressions for the data of the entire 10 Myr, identical to the black dashed lines shown in Fig. 3. The numbers in parentheses represent the slope of the regression line with one standard error. **b** probability density function of high latitude amplification factors. Histograms derive from 10,000 calculations of high latitude amplification factor with full propagation of errors. The blue and orange histograms represent results from the whole time series of the past 10 Myr and the period with an ice-free northern hemisphere (prior to 2.8 Ma), respectively. Blue and orange circles marked on the *x*-axis indicate the 68% percentile range.

The Pacific high latitude amplification is calculated to be 2.42 ± 0.64 (1*σ*), determined by the WPWP and high latitude data from North Pacific sites residing between 50°N to 55°N (Fig. 4b). If the SST data from the EEP are used instead of the WPWP, this high latitude amplification factor is 1.81 ± 0.52 (Supplementary Fig. 11). Given that the upwelled waters in the EEP carried the thermal signature of extratropical waters^44^, our following discussions of high latitude amplification are all relative to the WPWP.

Temperature gradient and amplification changes between the middle latitude and WPWP provide a test to examine whether our high latitude amplification determinations are biased by the availability of sites and data. Middle latitude sites with SSTs covering the last 10 Myr are available from both the North and South Pacific. Our results show that the warming in the middle latitudes was also amplified relative to the WPWP, with the middle latitude amplification factor of 1.57 ± 0.19 for the North Pacific, and 1.38 ± 0.09 for the entire Pacific (Supplementary Fig. 12). Over the past 10 Myr, the middle latitude amplification factors are smaller than high latitude amplification, consistent with our understanding that the amplification of warming is most pronounced in the high latitude regions (Fig. 3, Supplementary Fig. 12). The determination of middle latitude amplification lends independent support for the robustness of our analyses of the Pacific temperature patterns.

Over the last 10 Myr, the Earth has experienced pronounced climate changes. The atmospheric CO_2_ levels have varied between ~450 and 180 ppm^45^, and the equator-to-pole temperature gradient changed by more than 7 °C^12^ (Supplementary Fig. 9). In addition, ocean gateways changed and ocean circulation shifted^36^, and the cryosphere evolved substantially, with sea ice first appearing in the North Pacific at ~3 Ma^46^, followed by the initiation of major continental glaciation around 2.7 Ma^47^. Regardless of these changes, the high latitude amplification in the Pacific has remained constant, which argues for a robust physical mechanism constraining this relationship in the face of all these boundary condition changes over a 10 Myr.

The ice-albedo feedback is often regarded as a principal mechanism for high latitude amplification^48,49^. A substantial increase in ice-rafted debris was reported at our high-latitude Sites 882 and 887 around 2.75–2.7 Ma^46,50^, indicating the onset of significant Northern Hemisphere glaciation at this time. These sites used in our high-latitude SST compilation have been impacted by sea ice and ice sheets since ~2.75 Ma, and therefore can be used to evaluate the influence of the appearance of the Northern Hemisphere cryosphere on the magnitude of the high latitude amplification. Direct comparison between high latitude amplification before and after Northern Hemisphere glaciation (~2.7 Ma) is hampered by the small number of available high-latitude SSTs between 2.8 Ma and the present. We, therefore, opted for comparing the high latitude amplification factor for the Northern Hemisphere ice-free period (10–2.8 Ma) with that for the entire studied interval (10–0 Ma, Fig. 4).

The amplification factor was estimated to be 3.39 ± 0.84 (1*σ*) in the NH ice-free world (10–2.8 Ma) and larger than that of the entire 10 Myr (2.42 ± 0.64, Fig. 4b). This precludes the ice-albedo feedback as the primary driver of the high latitude amplification, consistent with the model results showing amplified polar warming without changes in snow and sea ice cover^7,9^. Processes other than surface albedo feedback, such as lapse rate and Planck feedbacks^51,52^, therefore are more likely to govern this amplification. However, it should be noted that this conclusion cannot be directly extrapolated to “Arctic amplification” since again, our analyses are restricted to the 50°N to 55°N North Pacific due to the lack of a comparable dataset from the Arctic Ocean. Nonetheless, the robustness of this Pacific high latitude amplification in paleoclimate data suggests it is caused by an intrinsic property of the climate system, which leads us to hypothesize that this high latitude amplification response pattern should show up in climate models. This hypothesis is tested, and implications are drawn in the following sections.

### High latitude amplification: reconstructions vs. models

**Fig. 5:**
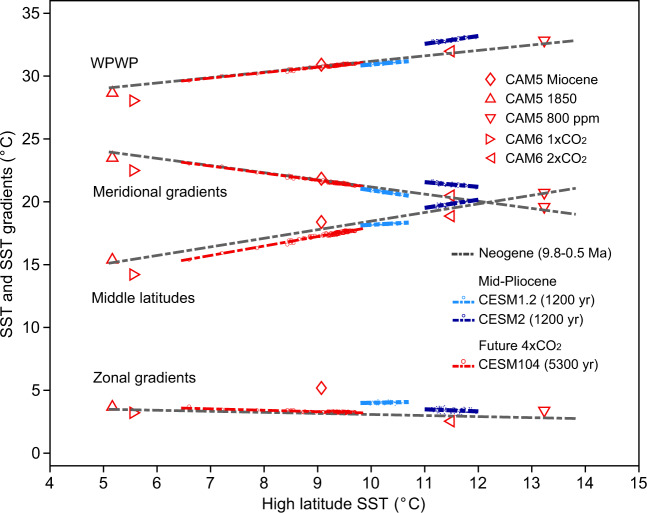
High latitude amplification from the past to the future. Meridional gradients are sea surface temperature (SST) differences between the Western Pacific Warm Pool (WPWP) and the high latitudes, and zonal gradients are SST differences between the WPWP to the Eastern Equatorial Pacific. Gray dash-dotted lines are regression lines of the 10-Myr SST data, the same as those represented in Fig. 3. Blue and red markers represent the model outputs generated by the Community Earth System Model (CESM). Light and dark blue circles represent the equilibrium mid-Pliocene simulations conducted with CESM version 1.2 (CESM1.2) and 2 (CESM2) at 400 ppm CO_2_, respectively^99^. Red circles represent the 5300-year SSTs generated by CESM 1.0.4 (CESM104) with abrupt atmospheric CO_2_ quadrupling (abrupt 4 × CO_2_) above pre-industrial levels^55^. Upward-pointing and downward-pointing triangles represent the ‘equilibrium’ temperatures for the preindustrial (1850) and 800 ppm of CO_2_ generated by CESM with version 5 of the Community Atmosphere Model (CAM5), respectively. Red diamonds represent the CAM5-derived temperatures in the middle Miocene with 400 ppm CO_2_. Right-pointing and left-pointing triangles represent CSEM2/CAM6-derived temperatures in response to present-day (1 × CO_2_) and instantaneously doubled CO_2_ (2 × CO_2_), respectively^60^. Dash-dotted lines indicate linear regressions of these data, and the associated statistical analyses are shown in Table 1. See the “Methods” section for the analyses of model-based temperatures.

With the well-defined patterns of SST changes described above, we can now compare them against climate simulations. We focus here on the National Center for Atmospheric Research (NCAR) Community Earth System Model (CESM) since it is an Intergovernmental Panel on Climate Change (IPCC)-class model participating in the Coupled Model Intercomparison Project Phases 5 and 6, and it has been widely applied in paleoclimate studies. The recent versions of CESM have shown promise in reproducing the Pliocene^53^ and early Eocene^54^ climates with a lower meridional temperature gradient than today. CESM run for preindustrial and modern conditions reproduce modern SST distributions (Fig. 5). It similarly produces SSTs and SST gradients for the equilibrium mid-Pliocene paleoclimate simulations, and for long equilibrated future higher CO_2_ simulations, that lie well within the range defined by our paleoclimate data (Fig. 5, Supplementary Figs. 13, 14 and Supplementary note 3).

In terms of Pacific high latitude amplification, the Neogene and the 5300-year transient coupled CESM simulations^55^ lie along the same regression lines, and these slopes are equivalent to that obtained from the mid-Pliocene CESM1.2 simulation (Fig. 5, Table 1). The broad agreement between the Pacific amplification patterns from data on millennium to tens of millions of years timescale suggests that the millennium-scale climatic processes towards the past or future equilibrium climate work generate similar amplification in the North Pacific, such as a coupled atmospheric and oceanic heat transport^56^.

**Table 1:** Statistical analyses of the linear regressions shown in Fig. 3 and 5

Regression	Time periods	Slope	SE	*T*	*P*	*R* squared	Regression method
WPWP vs. High latitudes	Neogene (9.8–0.5 Ma)	0.43	0.09				York
	Mid-Pliocene CESM1.2 1200 yr	0.38	0.04	9.3	9.1E−16	0.43	OLS
	Mid-Pliocene CESM2 1200 yr	0.62	0.04	14.4	3.1E−27	0.65	OLS
	CESM104 160 yr	0.50	0.07	7.3	5.7E−06	0.81	OLS
	CESM104 5300 yr	0.43	0.01	51.0	6.2E−75	0.96	OLS
Middle vs. High latitudes	Neogene (9.8–0.5 Ma)	0.68	0.09				York
	Mid-Pliocene CESM1.2 1200 yr	0.25	0.03	7.3	3.6E−11	0.31	OLS
	Mid-Pliocene CESM2 1200 yr	0.65	0.04	16.6	3.7E−32	0.71	OLS
	CESM104 160 yr	0.66	0.10	6.5	1.9E−05	0.77	OLS
	CESM104 5300 yr	0.75	0.02	47.6	6.0E−72	0.96	OLS
Zonal gradient vs. High latitudes	Neogene (9.8–0.5 Ma)	−0.08	0.03				York
	Mid-Pliocene CESM1.2 1200 yr	0.09	0.05	1.7	9.2E−02	0.02	OLS
	Mid-Pliocene CESM2 1200 yr	−0.16	0.07	-2.4	1.7E−02	0.05	OLS
	CESM104 160 yr	−0.19	0.08	-2.4	3.3E−02	0.31	OLS
	CESM104 5300 yr	−0.11	0.01	13.1	1.4E−23	0.62	OLS

The ordinary least-squares (OLS) regression and York Regression were used. *T* and *P* represent *t*-statistic and *p*-value for the regression slope, respectively. Since the slope and standard error (SE) for the Neogene data are estimated from 10,000 values generated by the Monte Carlo method, the *T*, *P*, and *R* squared values are not available and thus left blank. Zonal gradients here are sea surface temperature differences between the Western Pacific Warm Pool (WPWP) and the Eastern Equatorial Pacific.

**Fig. 6:**
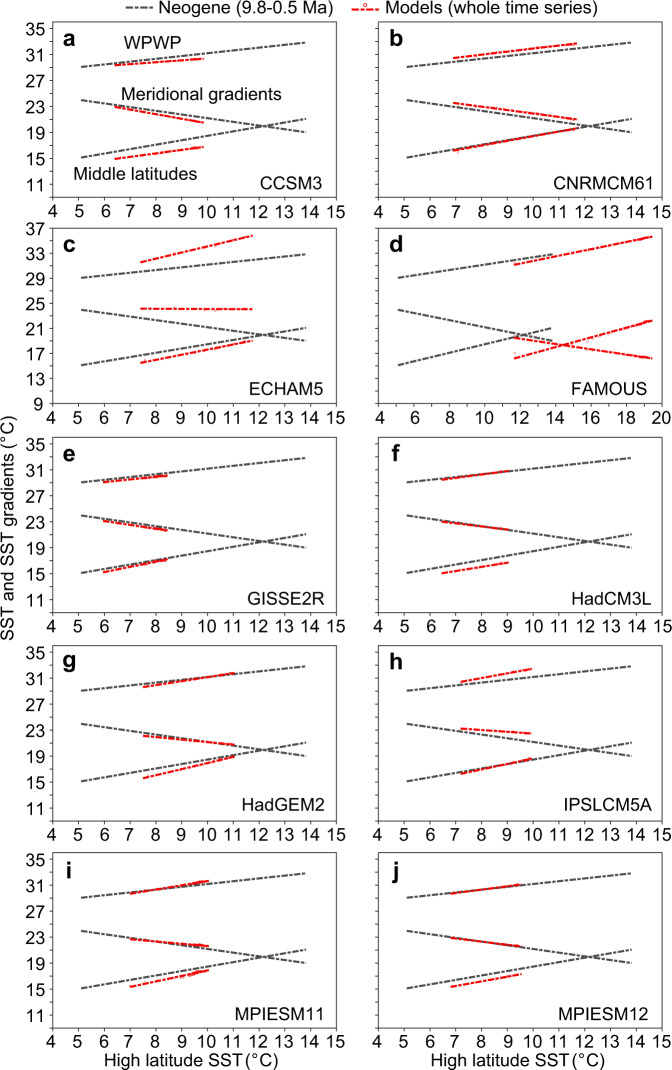
Comparing high latitude amplifications determined by the Neogene data to climate models. **a**–**j** show the comparison between the Neogene and future projection (millennial-length simulations with abrupt 4 × CO_2_) from CCSM3, CNRMCM61, ECHAM5, FAMOUS, GISSE2R, HadCM3L, HadGEM2, IPSLCM5A, MPIESM11, and MPIESM12, respectively. Meridional gradients are sea surface temperature (SST) differences between the Western Pacific Warm Pool (WPWP) and the high latitudes. Gray dash-dotted lines represent linear regressions of the 10-Myr SST data, the same as those shown in Fig. 3 and 5. Small red circles represent the results from each model output, and red dash-dotted lines represent linear regressions of these data. The slopes of the these linear regression are presented in Supplementary Table 3.

Besides CESM, we also compared our Neogene data with other millennial-length climate models^55^ and identified that majority of these models reproduce the middle and high latitude amplification more or less equally well except CCSM3, ECHAM5, and FAMOUS (Fig. 6, Supplementary Note 4 and Supplementary Fig. 15), suggesting they represent the physics of amplification correctly, but the more sensitive models exhibit a closer match to the range of high latitude SSTs over the past 10 Myr (Fig. 6 and Supplementary Table 4), given that the reconstructed late Miocene CO_2_ levels are ~400 ppm^45^ and lower than those (4 × CO_2_, 1120 ppm) in future climate simulations. Indeed, much of the recent progress in paleoclimate simulations in reproducing past warm climates appears to be due to increased climate sensitivity^54^. The warming pattern that emerged from this analysis can be used as a constraint on climate models and in this analysis, however preliminary, it is the models that are more sensitive and more accurately reproduce this pattern.

Importantly, regardless of the actual mechanism, the 10-million-year records (Fig. 5) presented here pave a possible path for our future high-latitude ocean regions, suggesting they will warm ~2.4 times as much as the WPWP SSTs. Arctic warming relative to WPWP is likely to be slightly higher than this value (Supplementary note 5, Supplementary Table 3, and Supplementary Fig. 16), consistent with an Arctic amplification factor of 2.2–2.4 relative to the global mean predicted in the Fifth Assessment Report of the IPCC^1^. These results clearly identify a specific amplification fingerprint for past warming which is not a strong function of time, boundary conditions, or greenhouse gas forcing trajectory^45^ thus enabling a ready comparison with climate model results. The technique of ordinating temperatures and temperature gradients by high-latitude temperatures should be broadly applicable across many regions and time intervals (Fig. 5).

Evaluating models based on how well they reproduce the amplification fingerprint can be a useful test of the model’s fidelity. It also might provide some idea of the magnitude of the “pattern effect” in our present climate; that is, the impact of the spatial pattern of surface warming on radiative feedbacks^57,58^, which is intimately linked with estimating climate sensitivity^59^. We can, however, derive a second constraint from the data shown in Fig. 5 by examining the range of SSTs themselves, which span high latitude SSTs of 5–12 °C, and comparing against climate models results across the range of forcings. In the case of various implementations of CESM, this comparison would be revealing. A CESM Miocene paleoclimate simulation with a CAM5 atmosphere (with an Equilibrium Climate Sensitivity or ECS of 4.1 °C/doubling) and at 400 ppm CO_2_ in the center of the range of CO_2_ proxy estimates, only warms half as much as data suggest at high latitudes (Fig. 5). A near-modern CESM/CAM5 simulation, with 800 ppm CO_2_ reproduces the full temperature range, which indicates that some substantial radiative forcing is still missing from the Miocene simulations or that the Miocene CESM sensitivity was too low. The just released CESM2/CAM6 model^60^, which has an ECS of 5.4 °C/doubling, is nearly as warm at high latitudes at only 569.4 ppm CO_2_ utilizing otherwise modern boundary conditions. These comparisons suggest that either the ECS value in CESM/CAM5 is too low for the Miocene, or other factors are important in driving the Miocene warmth. These other factors could include slow feedback from continental ice sheets or non-CO_2_ greenhouse gasses or aerosols or reorganizations of paleo-topography and continental configuration^61^. Regardless, discriminating between those options requires significant progress in understanding the CO_2_ levels during the late Miocene.

## Methods

### Age models

The age model of Site U1488 is based on integrated biostratigraphy and magnetostratigraphy^24^ established on Hole U1488A calibrated to the Geological Time Scale 2012 (GTS2012)^62^. Miocene age model of Site 806 was established from an existing biostratigraphy^63^ converted to the GTS2012 timescale by this study (Supplementary Table 1). The chronology of Site 1143 was also updated from the original publication^18^, using a recently published astronomically tuned age model (8.98–0 Ma)^64^, and the first occurrence of *Neogloboquadrina acostaensis* and *Discoaster neohamatus* observed at 465.8 and 488.79 m^65^, corresponding to 9.83 and 10.52 Ma, respectively. The chronology of Site 1388 was updated based on a revised biomagnetostratigraphic age model derived from Backman et al.^66^. The age model of other sites is based on their references which were already tuned to the GTS2012 timeframe^12^.

### Lipid biomarker analysis

304 of total lipids were extracted from 20 to 60 g of freeze-dried and crushed sediments of IODP Expedition 363 sites U1488, U1489, and U1490 with a mixture of dichloromethane and methanol (9:1, V/V) using an accelerated solvent extractor (ASE 350, DIONEX). Total lipid extracts were separated into aliphatic, aromatic, and polar fractions by silica-gel chromatography using hexane, dichloromethane, and methanol as respective eluents. The polar fraction containing tetraethers was dissolved in hexane:isopropanol (99:1, v/v), passed through 2.7 μm glass microfiber filter, and then analyzed by an Agilent 1260 series high-performance liquid chromatography (HPLC) coupled with Agilent 6120 series atmospheric pressure chemical ionization—mass spectrometry (APCI-MS) following the methodology of Becker et al.^67^ to measure the relative abundance of glycerol dialkyl glycerol tetraethers (GDGTs). An in-house laboratory standard was measured every five samples to check the reproducibility of the TEX_86_ values. TEX_86_ values were calculated according to Schouten et al.^68^, and the analytical precision is ±0.015 TEX_86_ unit based on long-term TEX_86_ measurements of the internal standard. The aromatic fractions containing ketones were dissolved in toluene and analyzed on an Agilent 7890B gas chromatography (GC) equipped with a 60 m DB-1 capillary column (0.25 mm ID, 0.25 mm film) and a flame ionization detector (FID) to measure the relative abundance of C_37:3_ and C_37:2_ alkenones. An in-house alkenone standard was measured every five samples to check the reproducibility of the ${{{{{{\rm{U}}}}}}}_{37}^{{{{{{\rm{K}}}}}}^{\prime} }$ values. ${{{{{{\rm{U}}}}}}}_{37}^{{{{{{\rm{K}}}}}}^{\prime} }$ values were calculated according to Prahl and Wakeham^69^, and then converted to SSTs using the Bayesian B-spline regression (BAYSPLINE) calibration^70^. Multiple measurements of an internal standard indicate that analytical precision is ±0.01 ${{{{{{\rm{U}}}}}}}_{37}^{{{{{{\rm{K}}}}}}^{\prime} }$ unit. All organic geochemistry was performed at Texas A&M University.

There must be abundant lipid biomarkers to ensure accurate GDGT and alkenone measurements and therefore TEX_86_ and ${{{{{{\rm{U}}}}}}}_{37}^{{{{{{\rm{K}}}}}}^{\prime} }$ value determinations. Since Site U1488, U1489, and U1490 has low organic carbon content^24^, large amounts (~20–60 g) of sediments were, therefore, requested and analyzed. This practice resulted in a high abundance of GDGTs and alkenones in most samples. However, there were still a small number of samples that yielded low abundance. For example, GDGT-3 is the most analytically challenging compound due to its typical lowest relative abundance among all isoprenoid GDGTs. Therefore, only samples with the integrated peak area of GDGT-3 above 1000 on our HPLC–MS were used to calculate TEX_86_ values. For samples below the detection limit, we concentrated the samples to improve the intensity of GDGT-3. For instance, during the first round of measurements, one “challenging” sample (U1488 A-4H-3, 62–65 cm; 30.8 g sediments used) shows the peak area of GDGT-3 as 1320 (Supplementary Fig. 1a) when 20 out of 500 μL solution was injected, slightly above our HPLC–MS detection limit of 1000. When this sample was concentrated and analyzed again with a 20 out of 200 μL injection, the peak area of GDGT-3 was increased to 3503 (Supplementary Fig. 1b). Although the TEX_86_ values from these repeated measurements are almost undistinguishable (0.69 vs. 0.70) (Supplementary Fig. 1), this practice generated better chromatograms that potentially led to more reliable determinations of TEX_86_ values.

### Planktonic foraminiferal Mg/Ca measurements

About 20 specimens of planktonic foraminifer *T. sacculifer* (without final sac-like chamber) were picked from the 250 to 300 μm size fraction of samples from Site 806 at the University of Massachusetts-Amherst. Tests of *T. sacculifer* were crushed and cleaned based on the cleaning methodology of Barker et al.^71^ (with the reductive step omitted) before being leached and diluted with nitric acid. Trace elements were then analyzed on the dissolved samples by a Thermo Element inductively coupled plasma mass spectrometry (ICP-MS) at the University of Southampton following Henehan et al.^72^. Mg/Ca precision was ±4% at two standard deviations based on repeat measurements of consistency standards measured at the same time as the unknowns. Al/Ca, a measure of the effectiveness of clay removal, was <100 μmol/mol in all but one sample (mean ~16 μmol/mol), confirming clay removal was adequate. The sample with an elevated Al/Ca of 150 μmol/mol was not obviously elevated in Mg/Ca and so was retained for completeness.

### Compilation of Pacific SST data

We compiled TEX_86_, ${{{{{{\rm{U}}}}}}}_{37}^{{{{{{\rm{K}}}}}}^{\prime} }$, and Mg/Ca data of the Pacific sites for the past 10 Myr^18–20,40^. The ${{{{{{\rm{U}}}}}}}_{37}^{{{{{{\rm{K}}}}}}^{\prime} }$ records from the eastern equatorial Pacific, middle latitudes (30°–50°), and high latitudes (>50°) over the past 10 Myr were compiled by Herbert et al.^12^ (Supplementary Table 2). For consistency, these SST estimates were recalculated using the TEX_86_ and ${{{{{{\rm{U}}}}}}}_{37}^{{{{{{\rm{K}}}}}}^{\prime} }$ calibrations described below. The lack of continuous SST records from the southern high latitude over the last 10 Myr limits our ability to evaluate the high-latitude amplification in the Southern Hemisphere.

### Paleogeography of study sites

Paleo-latitude and -longitude of sites shown in Fig. 1 were calculated using the GPlates software (http://www.gplates.org). Rotations and coastlines were obtained from Matthews et al.^73^, and the global continent–ocean boundary and spreading ridge dataset were from Müller et al.^74^. All sites remained in their large area of geographical feature (e.g., WPWP, EEP, middle latitudes, and high latitudes) for the entire studied interval.

### GDGT distributions

Most of the samples from Sites U1489 and U1490 are either below the detection limit of GDGTs or did not pass the screening tests described below, thereby limiting our interpretations of GDGTs as reflecting SSTs to Site U1488 only. Among the 109 samples taken from Site U1488, 11 were collected as the “squeezed cake” on board (samples after pore-water extractions) and 98 were sampled onshore at the Gulf Coast Repository of the IODP with a resolution of one sample per 100 kyr. Two samples did not have a sufficient abundance of GDGTs for the TEX_86_ determination. For the remaining 107 samples, we applied a series of screening methods. These approaches include ∆Ring Index (RI), the difference between measured RI and predicted RI from the global core-top TEX_86_–RI relationship^75^, to detect the samples affected by non-thermal factors. Based on the absolute value of ∆RI < 0.6, 12 samples were excluded to estimate TEX_86_–SST. The remaining 95 samples passed all other tests described below. The GDGT-2/crenarchaeol ratios (≤0.4) and methane index (≤0.5) were below their threshold values, indicating no substantial contribution of methanotrophic archaea to the GDGT pool^76,77^. GDGT-0/crenarchaeol ratios are <1.2 and %GDGT-0 values are <51, which reveals no major influence of methanogenic archaea^78^. GDGT-2/GDGT-3 ratios are <6.1, ruling out the major influence of deep-water-produced GDGTs^28^. %GDGT-2 values are low (<38), also suggesting that GDGT distributions are suitable for TEX_86_–SST^79^. The only exception is the branched and isoprenoid tetraether (BIT) index, presumably representing the relative abundance of the soil-derived GDGTs over marine GDGTs^80^. Among the 95 samples, 81% of them have BIT values >0.3, 38% have BIT values >0.4, and 13% have BIT values >0.5. However, a number of studies have shown that high BIT might not faithfully reflect soil inputs^81^ because of the sedimentary in-situ production of branched GDGTs^82^ that does not necessarily affect TEX_86_. For example, the reported Eocene and Oligocene BIT values from open oceans average 0.27 ± 0.19, much larger than the modern core-tops (0.03 ± 0.03)^83^. When TEX_86_ and BIT index of Site U1488 were cross-plotted, no significant correlation was identifiable (Supplementary Fig. 2). This observation, combined with the low C/N ratio throughout most of the site (mean value around 10), strongly suggests the predominance of marine production of the organic matter^24^. Consequently, the samples with relatively high BIT values but passing the Ring Index and all other tests were still included in our analyses.

### TEX_86_ calibration

The TEX_86_–SSTs from Sites U1488, 1143, 806, and 850 were estimated using the BAYSPAR calibration^30^. BAYSPAR calibration accounts for the spatial variations of TEX_86_–SST relationships, and paleo-latitude and -longitude of Sites U1488, 1143, 806, and 850 with 0.5 Myr interval were used in BAYSPAR calibration to produce SST estimates and the associated uncertainties.

### ${{{{{{\rm{U}}}}}}}_{37}^{{{{{{\rm{K}}}}}}^{\prime} }$-SST estimates

The ${{{{{{\rm{U}}}}}}}_{37}^{{{{{{\rm{K}}}}}}^{\prime} }$-SSTs from Sites U1488, 882, 883/884, 887, 1208, 1021, 594, 1125, U1338, 846, 850 were calculated using a recently published Bayesian B-spline approach (BAYSPLINE)^70^ which better captures the nonlinear behavior as ${{{{{{\rm{U}}}}}}}_{37}^{{{{{{\rm{K}}}}}}^{\prime} }$ index approaches 1 and allows a slightly higher maximum, but the calibration is not anchored by data at those hotter temperatures. To reflect the fact that when SST reaches ~29 °C, within the analytical uncertainty, ${{{{{{\rm{U}}}}}}}_{37}^{{{{{{\rm{K}}}}}}^{\prime} }$ reaches 1^14,84^, we utilized a prior standard deviation of 5 °C when ${{{{{{\rm{U}}}}}}}_{37}^{{{{{{\rm{K}}}}}}^{\prime} }$ > 0.9, 3 °C when ${{{{{{\rm{U}}}}}}}_{37}^{{{{{{\rm{K}}}}}}^{\prime} }$ > 0.95 and 2 °C when ${{{{{{\rm{U}}}}}}}_{37}^{{{{{{\rm{K}}}}}}^{\prime} }$ = 1. For Site U1488, 109 samples have measurable alkenones for ${{{{{{\rm{U}}}}}}}_{37}^{{{{{{\rm{K}}}}}}^{\prime} }$ determinations. Almost all of the ${{{{{{\rm{U}}}}}}}_{37}^{{{{{{\rm{K}}}}}}^{\prime} }$ values reach their maximum value of 1 prior to 3 Ma, and thus ${{{{{{\rm{U}}}}}}}_{37}^{{{{{{\rm{K}}}}}}^{\prime} }$-SST is mostly pinned to the maximum value (~29 °C) between 10 and 3 Ma (Fig. 2). In the high-latitude North Pacific, reported ${{{{{{\rm{U}}}}}}}_{37}^{{{{{{\rm{K}}}}}}^{\prime} }$ values between 2.8 and 0 Ma were available from only one Site, Site 882, and from two independent studies: Martínez-Garcia et al.^85^ and Yamamoto and Kobayashi^86^. ${{{{{{\rm{U}}}}}}}_{37}^{{{{{{\rm{K}}}}}}^{\prime} }$ values reported by Martínez-Garcia et al.^85^, where the relative abundance of alkenones was measured by gas chromatography chemical ionization mass spectrometry (GC–CIMS) aiming to detect compounds with much lower concentrations^87^, were inconsistent with those reported by Yamamoto and Kobayashi^86^, where alkenones were measured by the more traditional GC-FID. The dataset obtained from Yamamoto and Kobayashi^86^ suffers from a lower resolution over Pliocene and Pleistocene with only 15 data points after 2.8 Ma. Nevertheless, these data are sufficient for our binning within 0.2 Myr time window and not systematically biased toward either glacial or interglacial periods (Supplementary Fig. 7). In our compilation, we opted for the ${{{{{{\rm{U}}}}}}}_{37}^{{{{{{\rm{K}}}}}}^{\prime} }$ record reported by Yamamoto and Kobayashi^86^, because a higher alkenone abundance of their samples (averaged at ~0.08 μg/g) minimizes the potential biases^86,88^. For example, it has been observed that stronger absorption of C_37:3_ alkenones onto the surface of the capillary column when alkenone abundance was low^89^, which could lead to warm bias of ${{{{{{\rm{U}}}}}}}_{37}^{{{{{{\rm{K}}}}}}^{\prime} }$-SST. In addition, Site 882 samples with low alkenone concentrations could be more subject to laterally transported alkenones from subtropical regions^90^, which would again cause a warm bias of ${{{{{{\rm{U}}}}}}}_{37}^{{{{{{\rm{K}}}}}}^{\prime} }$-SST. Besides reducing the warm biases, the ${{{{{{\rm{U}}}}}}}_{37}^{{{{{{\rm{K}}}}}}^{\prime} }$-SST record from Yamamoto and Kobayashi is more consistent with the ${{{{{{\rm{U}}}}}}}_{37}^{{{{{{\rm{K}}}}}}^{\prime} }$-SST values of nearby Sites 883/884 (Supplementary Fig. 7), which were also determined by a GC-FID method^12^.

Besides BAYSPLINE, we also evaluated the influence of alternative linear calibrations such as Conte et al.^14^ on our results and found that linear calibrations will not change our estimates of high-latitude amplification factors (see Supplementary note 1).

### Mg/Ca-SST estimates

Mg/Ca values of Site 806 were converted to SSTs using the core-top calibration for tropical *T. sacculifer* derived from Dekens et al.^91^: Mg/Ca = 0.31 exp 0.084 [SST + 0.048(∆CO_3_^2−^)] where ∆CO_3_^2−^ = [CO_3_^2−^]in situ−[CO_3_^2−^]_saturation_ which corrects for carbonate dissolution. A constant, modern ∆CO_3_^2−^ value of 10.5 μmol/kg was applied for Site 806 10-Myr data^91^ since the water depth of Site 806 during the late Miocene and Pliocene (<2672 m)^92^ was always shallower than the horizon where significant dissolution occurred in the Pacific (>2800 m)^93^. This is recently confirmed by a B/Ca study of benthic foraminifera from Site 806, which suggests that carbonates at Site 806 were not subjected to severe dissolution during the Plio-Pleistocene^23^.

The residence time of Mg and Ca in the ocean is about 13 and 1 Myr, respectively, which requires the consideration of possible changes of Mg/Ca in the seawater (Mg/Ca_sw_) when applying Mg/Ca thermometry on timescales of >1 Myr. To adjust the influence of varying Mg/Ca_sw_ on the Mg/Ca thermometry, the ratio between the past and modern Mg/Ca_sw_ value is used following previous studies^38,94^, and the calibration equation is expressed as: Mg/Ca = (Mg/Ca_sw_/Mg/Ca_ps_) × 0.31 exp 0.084 [SST + 0.048(∆CO_3_^2−^)] where Mg/Ca_sw_ is the past seawater Mg/Ca and Mg/Ca_ps_ is the present value (5.2 mol/mol). Since the simulated SH98 Mg/Ca_sw_ agrees well with the proxy data and covers the entire 10 Myr, we applied SH98 Mg/Ca_sw_ to calculate Site 806 Mg/Ca-SST presented in Fig. 2. Besides Dekens et al.^91^, alternative calibrations were also used to covert Mg/Ca to SST (see Supplementary note 1), and the estimated SSTs from different calibrations were shown in Supplementary Fig. 4.

Besides temperature, pH, and salinity also affect the incorporation of Mg into the calcite shell of planktonic foraminifera^95^. However, the pH effect is minimal for the Mg/Ca of *T. sacculifer*^95^ and therefore not included in our Mg/Ca–SST calculations. A salinity change of 1 practical salinity unit (PSU) would lead to a change in foraminiferal Mg/Ca by ~4% and consequently SST by ~0.5 °C^95^. The limited constraints on the Miocene–Pleistocene surface salinity of the WPWP restrict our ability to assess the bias of salinity variations on Mg/Ca–SST of Site 806, but this bias is likely to be within the SST calibration uncertainty given that the variation in the EEP surface salinity is <1.1 PSU for the past 10 Myr^96^ and the surface salinity change in the WPWP is less than that in the EEP during the last glacial maximum^95^.

### Stacking regional SST for the past 10 Myr and model outputs

Pacific SST records (Supplementary Figs. 5 and 8) were stacked to produce the composite records from four regions: WPWP, EEP, middle latitudes, and high latitudes (Supplementary Fig. 9). In each region, SST data were binned over 200 kyr at each site, with 50% overlap, from 10 to 0 Ma. 200 kyr was selected as our bin size since it is consistent with our typical data resolution and fits our goal of determining the long-term amplification of warming over the last 10 million years. This bin size limits our analyses to long timescales and precludes investigations of the high-latitude amplification on glacial–interglacial timescales. When higher-resolution records become available, different bin sizes should be used to test whether the amplification factors determined here (Fig. 3, Table 1) are subject to changes.

For each time bin, we calculated the mean at each site and the standard deviation of the SST around the mean and then averaged the mean SST at all sites in a region. Therefore, we obtained WPWP, EEP, and middle-latitude SST stack and high-latitude SST at the same time series (Supplementary Figs. 5 and 9). The standard error of averaged SST between sites was calculated and regarded as the standard error of the regional SST stack. The binning window we used is 200 kyr except for the high-latitude SST binning between 2.8 and 0 Ma, when there were only 15 data points available from Site 882. We, therefore, used a larger window (400 kyr) for binning the 2.8-million-year SST data at this site. High-latitude SST was obtained from a single site during some time periods (2.6–0.6, 4.8–4.5, 9.8–7.5 Ma) due to the limited number of sites with available ${{{{{{\rm{U}}}}}}}_{37}^{{{{{{\rm{K}}}}}}^{\prime} }$ data. It was impossible to calculate the standard error of averaged SST between sites for these time periods, and thus the median of the calculated standard error of high-latitude SST stack for other time periods was used.

In terms of model outputs, several areal boxes bracketing our study sites were chosen to represent the WPWP (140°–160°E, 0°–4°N), EEP (118°W–90°W, 4°S–4°N), middle latitudes (156°E–160°E, 34°N–38°N; 126°W–130°W, 38°N–40°N; 174°E–178°W, 42°S–46°S) and high latitudes (168°E–148°W, 50°–56°N). We used the area-weighted yearly SST to represent the regional SST. For the 1200-yr mid-Pliocene CESM1.2 and CESM2 simulations, we binned the SST outputs over a 20-yr binning window to remove the influence of El Niño-Southern Oscillation and Pacific Decadal Oscillation, and then yielded one SST time series for each region, which were presented in Fig. 5. In terms of the 5300-yr transient coupled CESM104 simulation, we obtained a time-series of SSTs at each region over the first 160-year using the 20-year binning method. Since the model outputs are temperature anomalies, we started the 160-year data off to fit the observed recent 160-year SSTs in the EEP, WPWP, and middle latitudes at the lowest high-latitude SST. We then used these SST references at each region and generated a time series of SST over the whole time series using the 100-year binning approach. The same stacking method was applied to other millennial-length model simulations with abrupt 4 × CO_2_ forcing (Fig. 6).

### Regression analyses

We use two regression methods in our linear regression analyses, ordinary least-squares (OLS) regression, and York Regression. For model outputs, we applied the OLS regression since these temperatures and ages are associated with minimal uncertainties. For the 10-Myr proxy data, we employed York Regression to estimate the relative temperature change, given that temperatures in both the horizontal and vertical dimensions have analytical errors and uncertainties arising from temperature calibrations used to convert indices to SSTs. One exception for the proxy data is the SST trend from 0.5 to 9.8 Ma (Supplementary Note 2 and Supplementary Fig. 10), which is calculated using the OLS regression rather than York Regression due to difficulties in precisely determining the age uncertainties.

### High-latitude amplification factor determinations

High latitude amplification is defined as high latitude SST changes relative to tropical SST changes in this study. It can be estimated by the low latitude SST regressed on high latitude SST or vice versa. Instead of regressing the high latitude SST on the low latitude SST, here we chose to regress the low latitude SST on the high latitude SST since the high latitude SST covering the past 10 Myr has a larger range relative to the WPWP. To calculate the high latitude amplification factor over the past 10 Myr, we performed a York Regression of high-latitude SST against WPWP and EEP SST with their standard errors. Using a Monte Carlo approach, we conducted 10,000 simulations of binned SST at each site by randomly sampling the binned SST within their 2 standard deviations. Following the stacking method, 10,000 iterations of WPWP, EEP, and high-latitude SST were obtained with their standard errors. Utilizing these data, we generated 10,000 realizations of the York Regression of the high-latitude SST against WPWP and EEP SSTs, respectively. Therefore, 10,000 slopes of the high latitude versus WPWP SST and high-altitude versus EEP SST, respectively, were derived. The inverse of the slope represents the high latitude amplification factor. The distribution of 10,000 amplification factors (high latitude relative to the WPWP) was plotted as the probability density function of the high latitude amplification factor (Fig. 4b). The amplification factor is 2.42 ± 0.64 (1*σ*), indicating that the high-latitude SST changes are amplified relative to the WPWP by a factor of 2.42 ± 0.64. Similarly, the York Regression between high-latitude SST and EEP SST shows that the high-latitude SST change is amplified relative to the EEP merely by a factor of 1.81 ± 0.52 (Supplementary Fig. 11).

To calculate the amplification factor derived from model outputs of the mid-Pliocene and abrupt 4 × CO_2_ simulations, we performed the OLS regression of high-latitude SSTs against WPWP (Figs. 5 and 6).

### Meridional SST gradient vs. high latitude SST

The relationship between meridional SST gradient and high-latitude SST was derived from the linear relationship between the WPWP/EEP and high-latitude SST regardless of the reconstructed, observed, or simulated SSTs. Taking the paleo-SSTs as an example, we first yielded a linear relationship between the WPWP SST (*y*) and high-latitude SST (*x*) (Fig. 3), which gives:

1 $$y=0.43x+26.86$$

Then, we set meridional temperature gradient (WPWP minus high-latitude SST) as a variable *Y*. According to Eq. (1), we can derive the relationship between meridional SST gradient and high-latitude SST, which is expressed by

2 $$Y=y-x=\left(0.43x+26.86\right)-x=\left(0.43-1\right)x+26.86$$

Applying this indirect approach to other time scales or scenarios (Figs. 5, 6, Supplementary Figs. 11 and 12), we obtained the relationship (Eq. (2)) between meridional SST gradient and high-latitude SST. These relationships were only used in a qualitative sense to show that the SST differences from the equator to high latitudes decrease in a warmer world.

### Middle latitude SST changes relative to the WPWP

Mid-latitude sites are available from both the north and south Pacific, with the corresponding modern study area from the north Pacific (156°E–160°E, 34°N–38°N; 126°W–130°W, 38°N–40°N) and south Pacific (174°E–178°W, 42°S–46°S) region. However, the SST data from the South Pacific are quite limited; for example, Site 1125 is the only available record between 10 and 2.8 Ma. Our results show that the warming in the middle latitudes was also amplified relative to the WPWP, with the middle-latitude amplification factor of 1.57 ± 0.19 for the North Pacific (Supplementary Fig. 12). The inclusion of the South Pacific data does not make a statistically significant change, with the value computed to be 1.38 ± 0.09 for the entire Pacific (Supplementary Fig. 12).

### Model outputs

CESM outputs used to compare with observations and reconstructions are from paleoclimate, modern, and future simulations. Miocene global paleoclimate simulation (with an ECS of 4.1 °C/doubling) was carried out for >2000 years utilizing CESM/CAM5 incorporating the Community Land Model (CLM4)^97^ and updated Miocene boundary conditions^98^, including lower albedo and different ocean circulation, and 400 ppm CO_2_, which is in the center of the range of CO_2_ proxy estimates. The “equilibrium” temperatures of modern and future simulations are derived from publicly available CESM/CAM5 and the recently released CESM2/CAM6 models. The equilibrium mid-Pliocene (3.205 Ma) simulations were carried out for 1200 years using the CESM1.2 (with an ECS of 4 °C/doubling) and CESM2 (with an ECS of 5.3 °C/doubling)^99^, and their boundary conditions follow the Pliocene Model Intercomparison Project Phase 2 (PlioMIP2), which utilizes 400 ppm CO_2_. CESM/CAM5 model outputs from the preindustrial (1850) and present-day (800 ppm CO_2_ forcing) runs are utilized in our comparison since they reproduce the full temperature range of paleoclimate data, and the corresponding netcdf files we used are E.1850_C5-TS_avg.nc and E.Mod_800_C5-TS_avg.nc. CSEM2/CAM6 provides temperatures in response to present-day (1 × CO_2_) and instantaneously doubled CO_2_ (2 × CO_2_). The millennial-length future simulations included in our study were obtained from Rugenstein et al.^55^ and these model outputs generated with abrupt 4 × CO_2_ forcing (Table 1 and Supplementary Table 4) were employed for our comparison since a CO_2_ quadrupling is requested by Coupled Model Intercomparison Projects 5 and 6 and represent the future CO_2_ levels on a multi-century time scale.

## Supplementary information

Supplemental Information

## Data Availability

The raw data (TEX_86_ and ${{{{{{\rm{U}}}}}}}_{37}^{{{{{{\rm{K}}}}}}^{\prime} }$ from Site U1488 and Mg/Ca from Site 806) generated by this study as well as the site-specific SST used for the SST stack of the WPWP can all be found at Figshare (10.6084/m9.figshare.21183769). The CESM2/CAM6 outputs with 1 × CO_2_ and 2 × CO_2_ scenarios are publicly available at 10.26024/zrad-5z41. The mid-Pliocene CESM2 model outputs are available through the Earth System Grid at 10.22033/ESGF/CMIP6.7675.

## Code availability

The NCAR CESM 1 model with CAM5 atmosphere is freely available from NCAR (http://www.cesm.ucar.edu/models/cesm1.0/).
