# Supplementary material for: Persistent high latitude amplification of the Pacific Ocean over the past 10 million years
Source: Nat Commun. 2022 Nov 27;13:7310. doi: 10.1038/s41467-022-35011-z (PMC9701799; doi:10.1038/s41467-022-35011-z)
Supplement: Supplementary file 1 — Supplemental Information [file 41467_2022_35011_MOESM1_ESM.pdf]

Supplementary Information for

Persistent high latitude amplification of the Pacific Ocean over the past 10 million years

Authors: Xiaoqing Liu[1,2], Matthew Huber[2], Gavin L Foster[3], Andrew Dessler[4], and Yi Ge Zhang[1*]

Affiliations:

[1]Department of Oceanography, Texas A&M University; College Station, Texas 77843, USA.

[2]Department of Earth, Atmospheric and Planetary Sciences, Purdue University; West Lafayette, Indiana 47907, USA.

[3]School of Ocean and Earth Science, University of Southampton, National Oceanography Centre Southampton; Southampton, SO14 3ZH, UK.

[4]Department of Atmospheric Sciences, Texas A&M University; College Station, Texas 77843, USA.

*Corresponding author. Email: yige.zhang@tamu.edu

This file includes:

Supplementary notes (note 1-5)

Supplementary figures (Fig. 1-16)

Supplementary tables (Table 1-4)

Supplementary note 1. Alternative calibrations

Alternative calibrations for $\mathbf{U^{K'}_{37}}$. $U^{K'}_{37}$ can also be converted to SST using the linear calibration such as Conte et al[1]. Since this linear calibration does not address the non-linear response of $U^{K'}_{37}$ to SSTs near the warmer end of the calibration dataset, we applied it to the extratropical sites to generate an alternative scenario as a measure of the robustness of our conclusions. The SST analyses derived from the linear calibration yielded a high latitude amplification factor of 2.45 ± 0.59, which is almost identical to 2.42 ± 0.64 based on the BAYSPLINE.

Alternative calibrations for Mg/Ca-SST. Several alternative calibrations are available, including a regional core-top calibration in the WPWP[2], a global core-top Bayesian calibration model for Mg/Ca (BAYMAG)[3], and a laboratory culture-based calibration[4]. Applying these calibrations and the adjustment of SH98 Mg/Ca_{sw} to Site 806 Mg/Ca resulted in different SST estimates (Supplementary Fig. 4). The calibrations including the dissolution correction, such as BAYMAG[3] and Dekens et al.[5], produce similar SST estimates (Supplementary Fig. 4a) which track the TEX_{86}-SST. In contrast, the calibrations without the dissolution correction[2,4] yield lower Mg/Ca-SST estimates (Supplementary Fig. 4a and 4b). Among these calibrations, we used Dekens et al.[5] to convert Mg/Ca of Site 806 to SST since this calibration corrects for carbonate dissolution and is largely based on data from the tropical Pacific.

Supplementary note 2. Linear SST trend over the past 10 Myr

Using the time-binning approach, we obtained a new time series of SST data at each site from 9.8 to 0.5 Ma (in 200-kyr window), and then performed the OLS regressions of the age against the mean SST to obtain the linear SST trend — the slope of the regression line. Although

the age uncertainties of some sites (Sites 594, 1125, 1208, 883/884, 887 and 846) have been reported by Herbert et al.[6], these uncertainties were obtained from assigned age uncertainties for the orbital stratigraphy, polarity sequence and biostratigraphy, which were treated as different constants at varying time intervals. Also, the age uncertainties of the other study sites are difficult to determine. Therefore, instead of using York Regression which requires the uncertainty of both x and y to be known, we applied the OLS regression to estimate linear SST trend from the proxy data. The linear SST trend over the past 10 Myr (Supplementary Fig. 10) shows amplified warming in the high latitude relative to the WPWP. The p-value were calculated in the linear regression to evaluate the significance of warming trend. The paleo-results suggest that except Sites 882 (p-value = 0.40) and 887 (p-value = 0.11) which were limited by the number of data, all other sites show significant warming in the Mio-Pliocene (Supplementary Fig. 10).

Supplementary note 3. Single-site relative to high latitude SST change

We applied York Regression to obtain specific-site SST relative to high latitude SST change between 9.8 and 0.5 Ma (Supplementary Fig. 13, 14 and 15). This is performed by using high-latitude SST as x-axis and binned SST of individual site as y-axis. In addition to the WPWP and middle latitude sites discussed earlier, Site 1146 was also included in this analysis because of the available Mg/Ca-SST data covering 10-5 Ma[2,7], although it does not reside in any of our climate zonation classifications (WPWP, middle latitude or high latitude). The reported data were adjusted to the modeled SH98 Mg/Ca_{sw} scenario[8] to be consistent with our Site 806 SSTs (Fig. 2). Similarly, we applied the OLS regression to the model outputs from mid-Pliocene simulations and abrupt quadrupled-CO_2 (4×CO_2) simulations and yielded specific-site SST change relative to high-

latitude SST change over the whole time series of the model simulation (Supplementary Fig. 13, 14 and 15).

Supplementary note 4. Data *vs.* models

In terms of the equilibrium mid-Pliocene simulations, the comparison between CESM1.2 and CESM2 reveals that the Pacific warming pattern derived from CESM2 agree better with the 10-Myr paleoclimate record than that from CESM1.2 (Fig. 5 and Supplementary Fig. 13), although CESM2 generates higher SST in the WPWP than the reconstructed one (Fig. 5). The Pacific high latitude amplification factor derived from the mid-Pliocene CESM1.2 simulation (2.68 ± 0.30) is nearly identical to that from the Neogene record (2.42 ± 0.64), substantially higher than the value (1.61 ± 0.11) from the CESM2 simulation (Fig. 5 and Table 1). For the site-specific relative to high latitude SST change, CESM1.2 simulation generated smaller values than CESM2 which yielded comparable values with those from the 10-Myr record in the WPWP and middle latitudes (Supplementary Fig. 13).

Here we also evaluate if millennial-length climate simulations with abrupt 4×CO_2 forcing[9] are capable to reproduce the middle and high latitude amplification pattern observed in the Pacific for the past 10 Myr. Besides CESM104 (Fig. 5), CNRMCM61, GISSER2, HadCM3L, and MPIESM12 also reproduce the observed middle and high latitude amplification well (Fig. 6 and Supplementary Table 3). Although the Pacific high latitude amplification factors derived from CCSM3, HadGEM2, IPSLCM5A, and MPIESM11 slightly deviate from the Neogene value, the temperature variations from these models are within the Neogene range. In contrast, the WPWP, mid- and high-latitude temperature changes from FAMOUS are larger than those observed for the last 10 Myr, which is expected due to its high climate sensitivity (8.55°C/doubling, Supplementary Table 3), despite that the magnitude of high latitude amplification derived from FAMOUS agrees

with the Neogene data. ECHAM5 also produces larger SST change in the WPWP than the 10-Myr record while the mid- and high latitude temperature changes from ECHAM5 are smaller compared with the 10-Myr record.

For the single-site relative to high latitude SST change (Supplementary Fig. 13, 14 and 15), all the models generate similar results to the Neogene data in the WPWP except ECHAM5 with higher values and CCSM3 with lower values. For the sites in the EEP, no model is capable of fully reproducing the Neogene records. For the mid-latitude sites, CCSM3 and HADCM3L produce lower SST changes than the Neogene data; CESM104 and ECHAM5 yield similar results to the Neogene data at four sites, and the other models do not completely reproduce the reconstructed records at all four sites (Supplementary Fig. 15). Overall, all the models except CCSM3, ECHAM5 and FAMOUS well reproduce the pattern of the middle and high latitude amplification found in the Pacific for the past 10 Myr.

Supplementary note 5. Pacific high latitude amplification *vs.* Arctic amplification

Arctic amplification (AA) factor is commonly defined by the ratio of the Arctic (67.5°N to 90°N) temperature change to the global average temperature change[10]. Here we evaluate the relationship between AA factor and Pacific high latitude amplification factor defined in our study, based on CESM/CAM5 and CESM/CAM6 simulations, and millennial-length simulations with abrupt 4×CO_2 forcing from different climate models[9]. We calculated CSEM/CAM5-derived surface warming in the year 2000 with the present-day CO_2 forcing or 800 ppm CO_2 forcing relative to the pre-industrial (PI), mid-Miocene relative to PI, and CSEM2/CAM6-derived surface warming from present day (1×CO_2) to instantaneously doubled CO_2 (2×CO_2) forcing (Supplementary Fig. 16). The comparison between different amplification factors shows that AA

factor is moderately larger than the Pacific high-latitude amplification factor, as expected (Supplementary Fig. 16). In addition, surface air temperature (tas) anomaly over the last 30 years of each simulation with abrupt 4×CO_2 forcing (Supplementary Table 4) reveal that Pacific high latitude amplification is weaker than AA in nine models except CCSM3 and FAMOUS, which retain larger high latitude amplification factor than other models. Based on these comparisons, the AA factor for the past 10 Myr, although it cannot be constrained due to lack of SST records from the Arctic Ocean, is likely to be slightly higher than 2.42 ± 0.64, the Pacific high latitude amplification factor obtained from the paleoclimate data. Similarly, simulations summarized in the Fifth Assessment Report of the IPCC have predicted that the Arctic will warm 2.2-2.4 times as fast as the global average at the end of 21st (2081-2100) century[3].

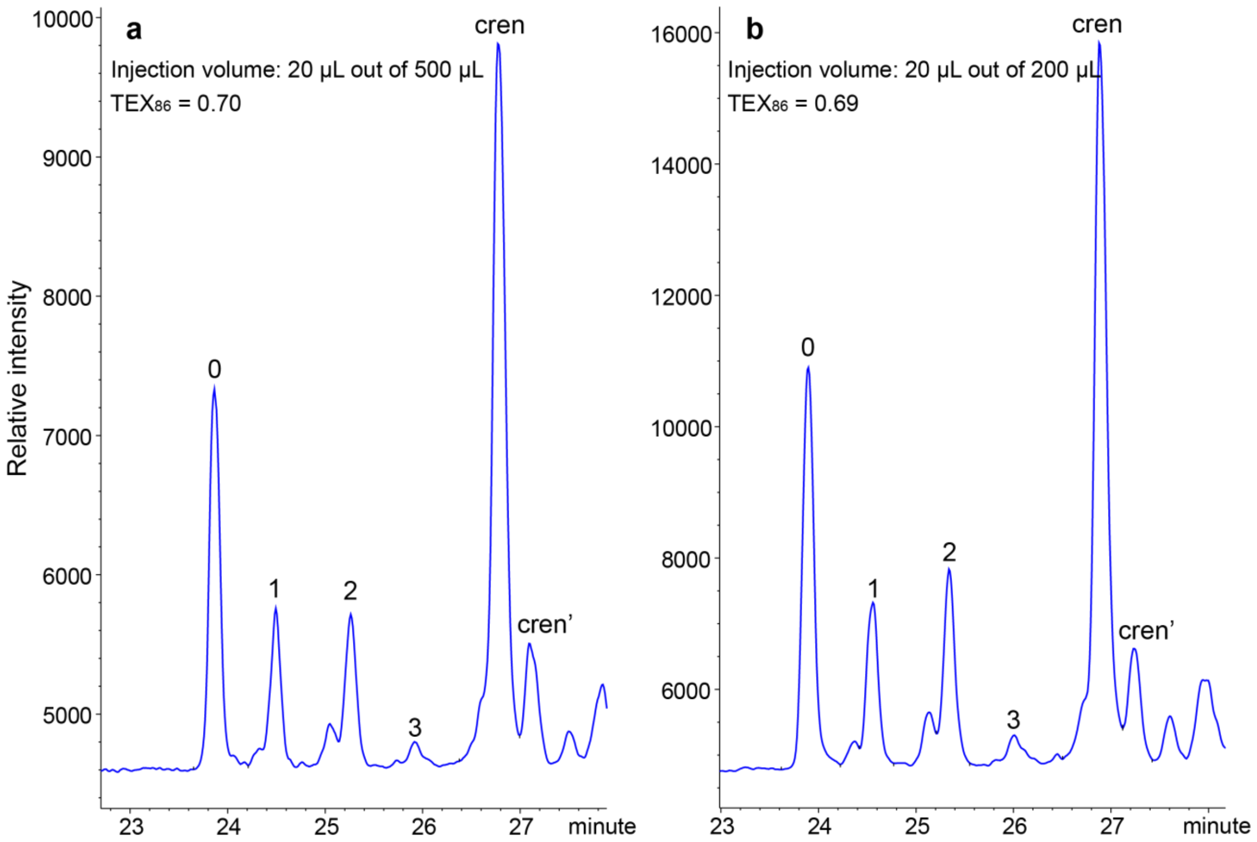

Supplementary Fig. 1. Chromatograms of a representative sample of Site U1488 to show the relative abundance of GDGTs determined by repeated measurements with different analyte concentrations. **a** and **b** are chromograms of one "challenging" sample (U1488A-4H-3, 62-65 cm) from a diluted (500 µL) and concentrated (200 µL) solution containing glycerol dialkyl glycerol tetraethers (GDGTs), respectively. 0, 1, 2, 3, cren and cren' indicate GDGT-0, GDGT-1, GDGT-2, GDGT-3, crenarchaeol, and crenarchaeol isomer. The injection volume on the high-performance liquid chromatography-mass spectrometry (HPLC-MS) and TEX_{86} values are also shown.

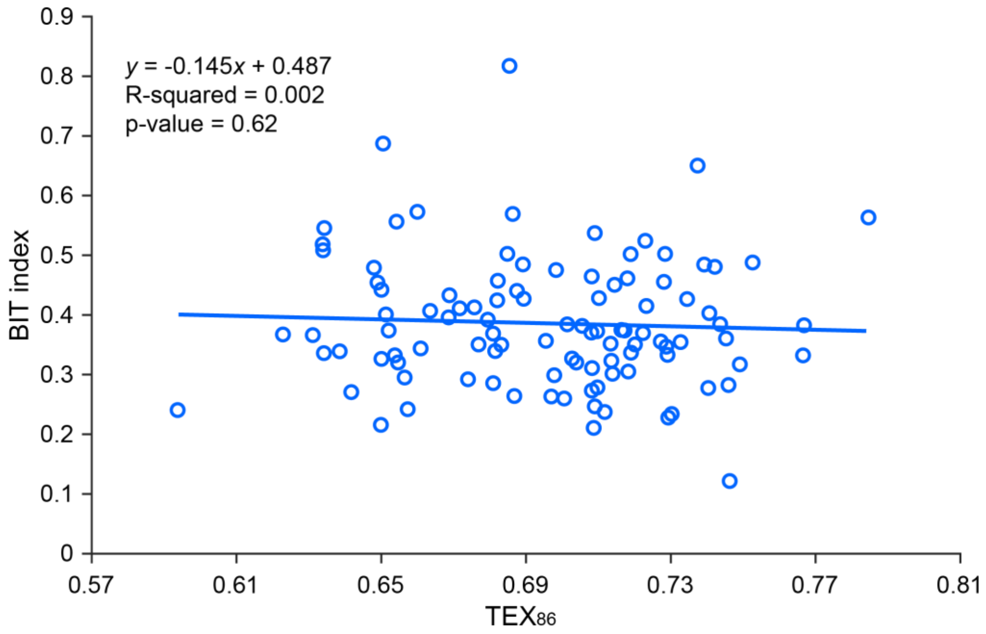

Supplementary Fig. 2. The cross plot between TEX_{86} and BIT index values of Site U1488. Blue line represents the ordinary least squares regression. The p-value for the slope is 0.62, indicating that there is no significant linear relationship between TEX_{86} and branched/isoprenoid tetraether (BIT) index at a significance level of 0.05.

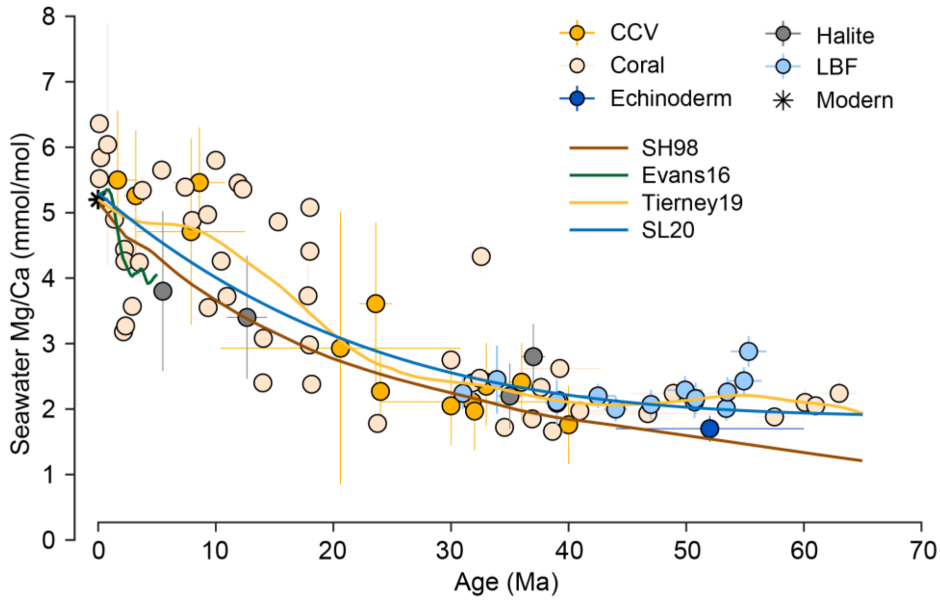

Supplementary Fig. 3. Different Seawater Mg/Ca scenarios over the Cenozoic. Mg/Ca values Orange, light pink, dark blue, gray and light blue circles represent Mg/Ca values measured from calcium carbonate veins (CCV), fossil corals, fossil echinoderms, halite fluid inclusions and large benthic foraminifera (LBF), respectively, which are obtained from the compilation done by Tierney et al[3]. Error bars show two standard errors of the mean. Brown, green, yellow and pink lines represent Mg/Ca_{sw} scenarios from Stanley and Hardie (SH98), Evans et al.[11] (Evans16), Tierney et al.[3] (Tierney19) and Sosdian and Lear[12] (SL20), respectively.

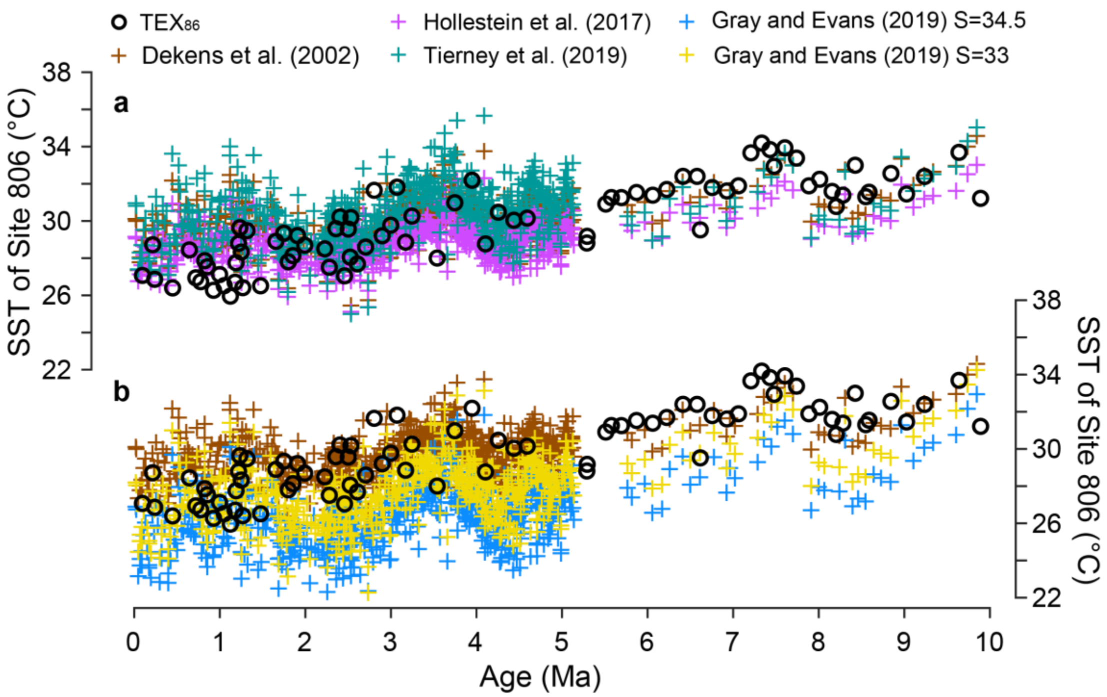

Supplementary Fig. 4. Site 806 Mg/Ca-SST using different calibrations, in comparison with TEX_{86}-SST of the same site. All Mg/Ca-based sea surface temperatures (SSTs) were corrected for seawater Mg/Ca changes based on SH98 scenario. **a**, Mg/Ca-SST using the calibrations of Dekens et al.[5] (brown), Hollestein et al.[2] (purple) and Tierney et al.[3] (with pH of 8.1 and salinity of 34.5, green). **b**, Mg/Ca-SST using the calibrations of Dekens et al.[5] (brown) and Gray and Evans[4] with salinity of 34.5 (blue) and 33 (yellow).

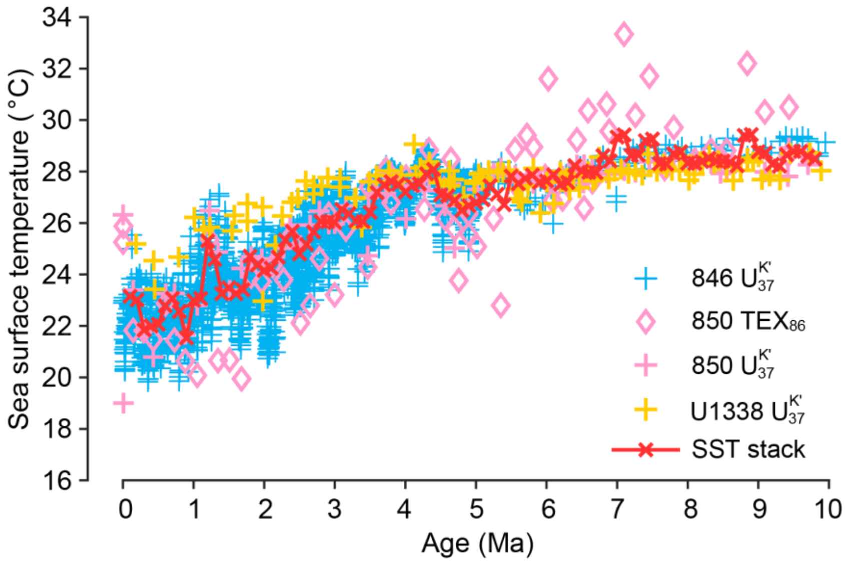

Supplementary Fig. 5. Sea surface temperature reconstructions of the Eastern Equatorial Pacific over the past 10 million years. Blue, pink, and yellow crosses show $U_{37}^{K'}$-derived sea surface temperature (SST) of Sites 846, 850 and U1338, respectively. Diamonds indicate TEX_{86}-SST of Site 850. The red line indicates the SST stack of the Eastern Equatorial Pacific calculated with $U_{37}^{K'}$-SST from Sites 846[6], 850[13] and U1338[14,15] and TEX_{86}-SST from Site 850[13].

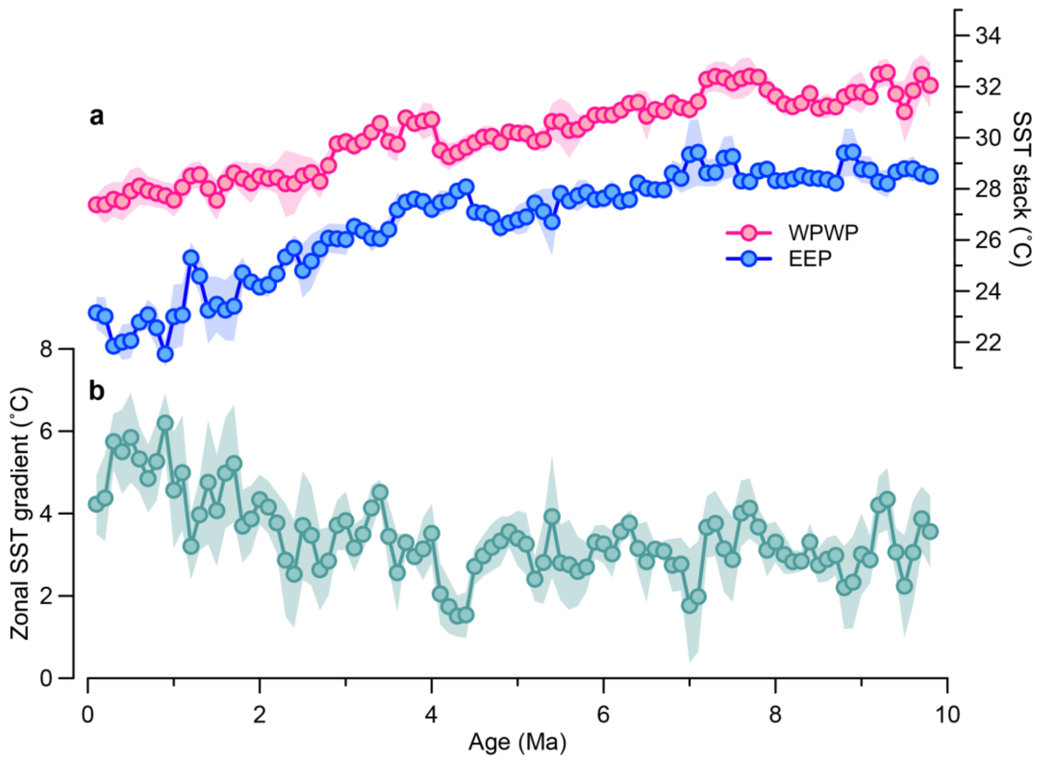

Supplementary Fig. 6. SST stack in the WPWP and EEP and zonal SST gradients of the Pacific Ocean for the past 10 million years. a, purple and blue circles represent sea surface temperature (SST) stacks of the Western Pacific Warm Pool (WPWP) and Eastern Equatorial Pacific (EEP), respectively. **b**, zonal temperature gradients from the WPWP to the EEP. Shadings indicate one standard error around the mean.

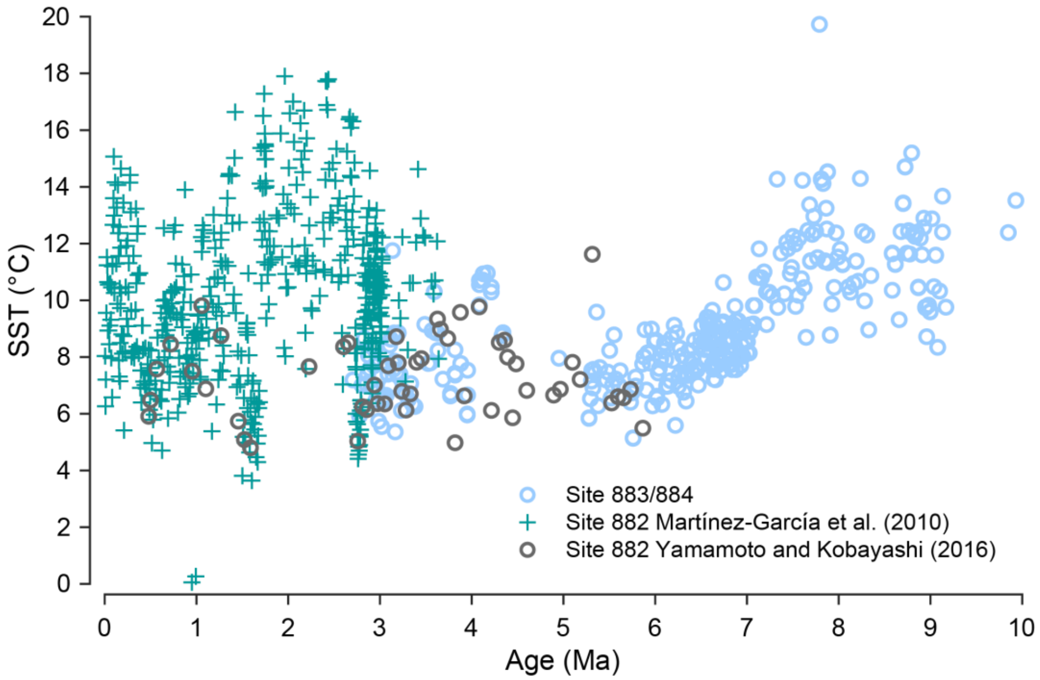

Supplementary Fig. 7. Comparing Sites 882 and 883/884 $U^{K'}_{37}$-SST from different sources. Blue circles show sea surface temperatures (SSTs) of Site 883/884[6]. Green crosses and gray circles show the SST of Site 882, with $U^{K'}_{37}$ values derived from Martínez-Garcia et al.[16] and Yamamoto and Kobayashi[17], respectively. $U^{K'}_{37}$-SSTs shown here are estimated using the Bayesian B-spline regression (BAYSPLINE) calibration[18].

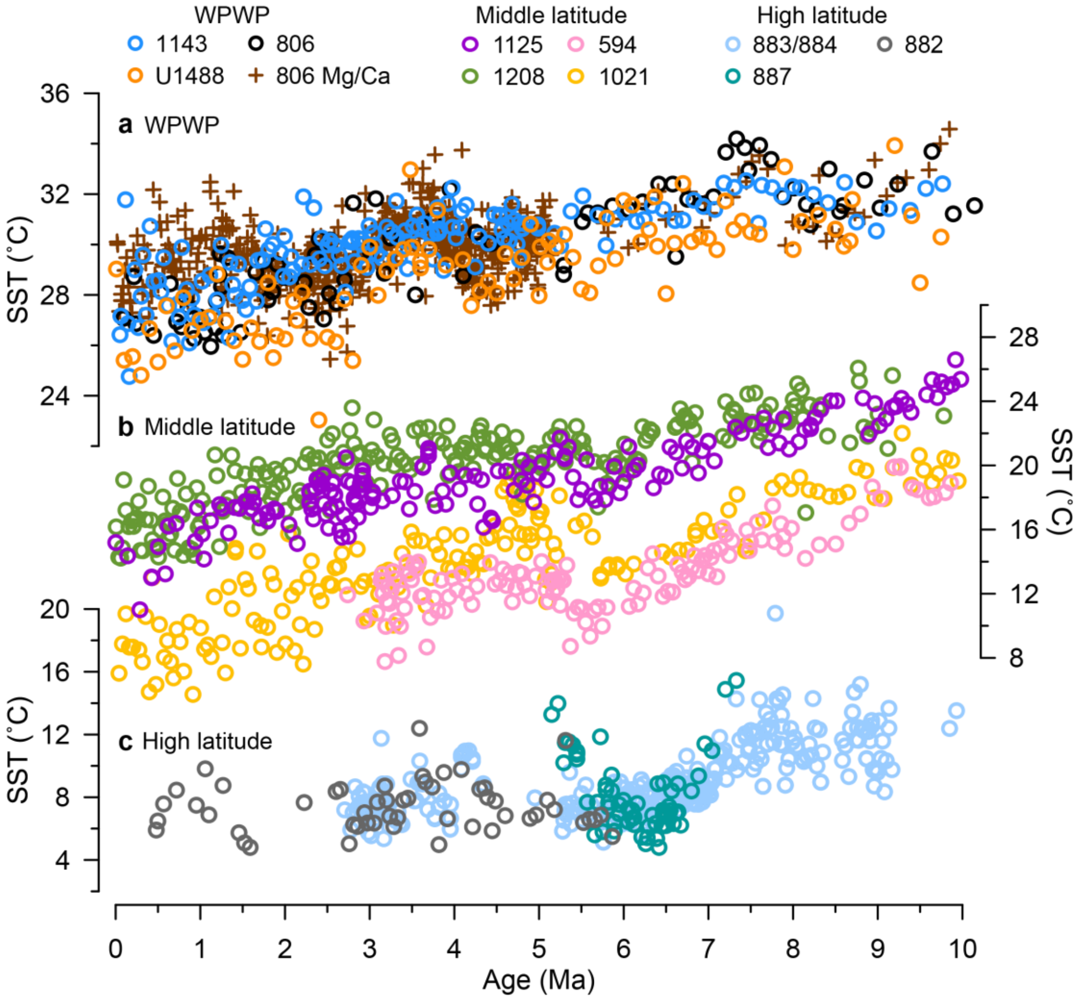

Supplementary Fig. 8. Individual SST records from the WPWP, middle latitudes and high latitudes. a, sea surface temperature (SST) compilation of the Western Pacific Warm Pool (WPWP). Cross denotes Mg/Ca-SST and circle represents TEX_{86}-SST. **b**, $U^{K'}_{37}$-SST compilation of middle latitudes, and $U^{K'}_{37}$ data of Sites 1208, 1021, 594, 1125 are derived from Herbert et al[6]. **c**, $U^{K'}_{37}$-SST compilation of high latitudes. $U^{K'}_{37}$ data of Sites 883/884 and 887 were obtained from Herbert et al[6]. $U^{K'}_{37}$ data of Site 882 were obtained from Yamamoto and Kobayashi[17]. Gray bar highlights the late Miocene cooling between 7 and 5.5 Ma. These SSTs were used to produce the regional SST stack presented in Supplementary Fig. 9.

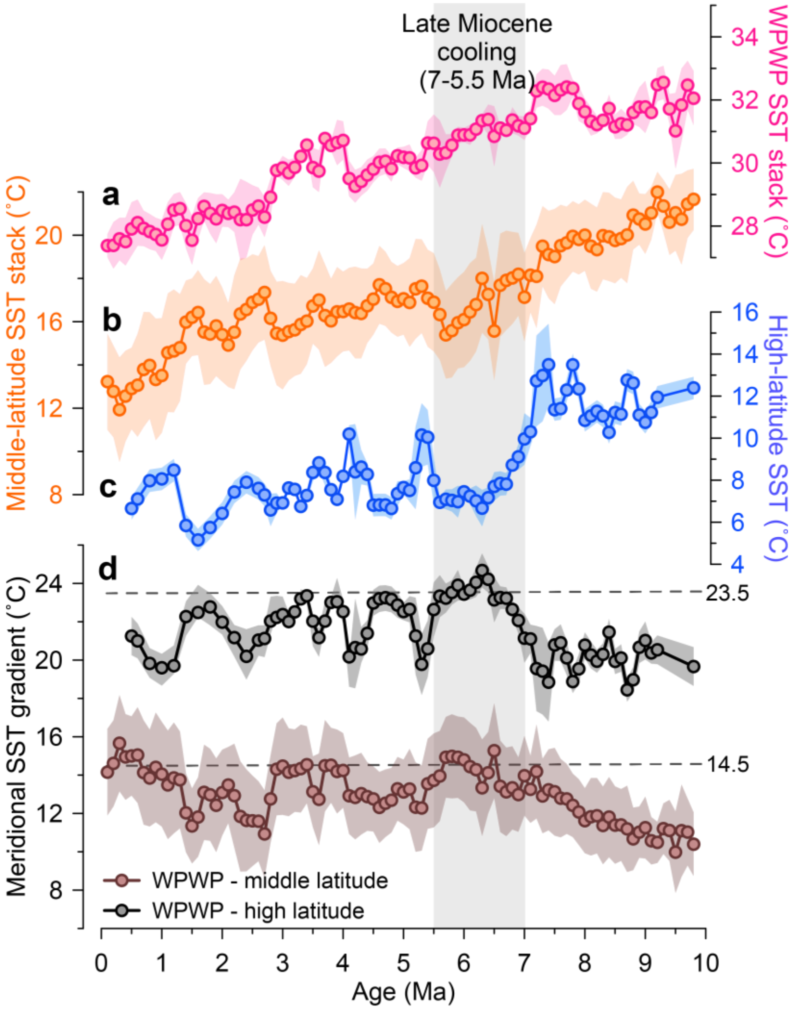

Supplementary Fig. 9. Pacific SST of the WPWP, middle latitudes and high latitudes, and meridional SST gradients over the past 10 million years. a, sea surface temperature (SST) stack of the Western Pacific Warm Pool (WPWP); **b**, mid-latitude SST stack. **c**, high-latitude SST. High-latitude SST rather than SST stack is used here since high-latitude SST is derived from a single site during some time intervals. **d**, meridional temperature gradient from the WPWP to middle latitude (brown circles) and from the WPWP to high latitude (gray circles). Dashed lines represent the modern-day meridional SST gradient. Shadings indicate one standard error around the mean. Gray bar highlights the late Miocene cooling between 7 and 5.5 Ma. Sites used to generate the WPWP and mid-latitude SST stack and high-latitude SST are seen in Supplementary Fig. 8.

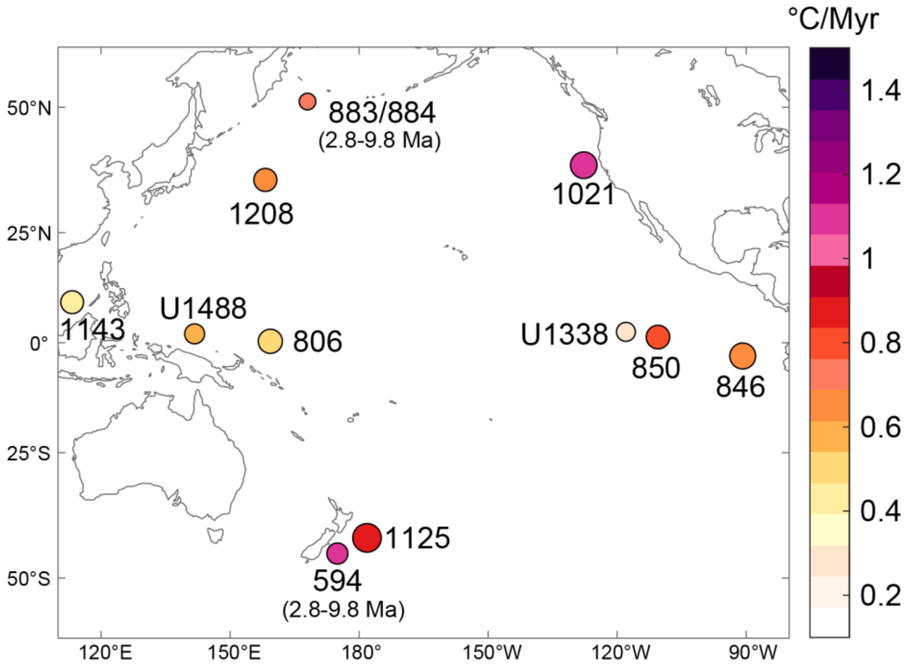

Supplementary Fig. 10. Linear SST trend from 0.5 to 9.8 Ma. Colors indicate values of the linear sea surface temperature (SST) trend in °C per million years (Myr), and larger circles represent greater t-statistic and better linear regression. The sites with age specification show the linear SST trend during the specific time interval when the SST data is available, and the other sites have the linear SST trend from 0.5 to 9.8 million years ago (Ma).

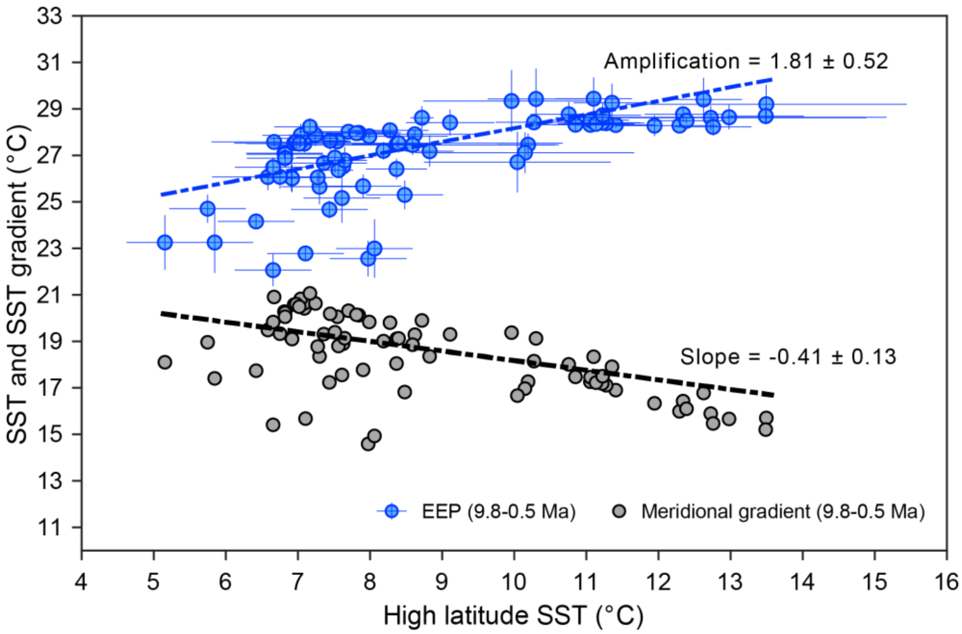

Supplementary Fig. 11. High latitude SST changes relative to the EEP for the past 10 Myr. Gray circles indicate meridional sea surface temperature (SST) gradients, that is, SST differences between Eastern Equatorial Pacific (EEP) and high latitudes. Error bars around blue circles indicate one standard error due to stacking. Blue dashed line shows York regression, and black dashed line represents the linear fit derived from the relationship between EEP and high-latitude SST.

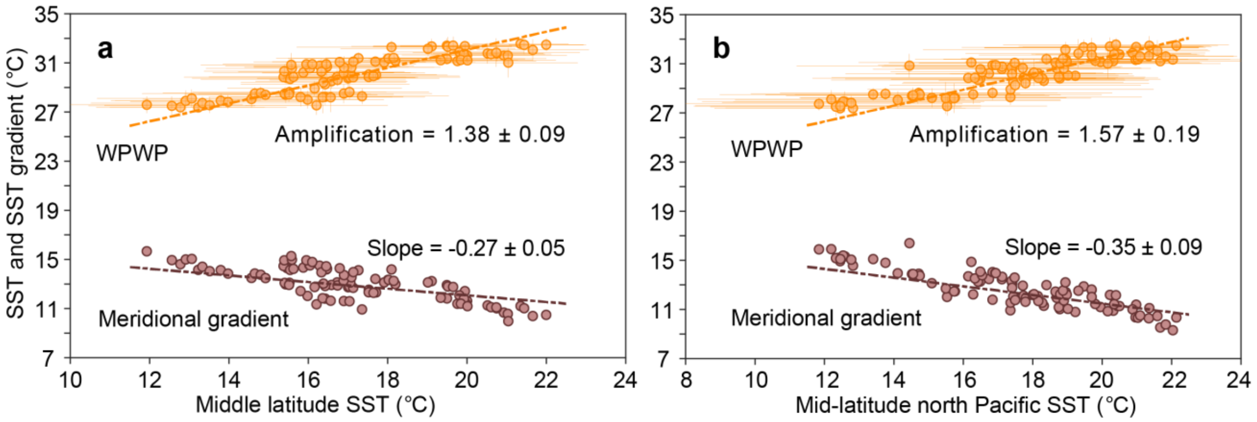

Supplementary Fig. 12. Middle latitude sea surface temperature changes relative to the Western Pacific Warm Pool for the past 10 million years. a, middle latitudes signify all sites and regions in the north and south Pacific. **b**, middle latitudes only include the sites and regions in the north Pacific. Error bars around orange circles indicate one standard error due to stacking. Orange dashed lines show York Regression, and brown dashed lines represent the linear fit derived from the relationship between Western Pacific Warm Pool (WPWP) and mid-latitude sea surface temperature (SST). Brown dashed lines indicate the regression of high latitude SST against SST gradients between the WPWP and middle latitudes (**a**) and middle latitude north Pacific (**b**).

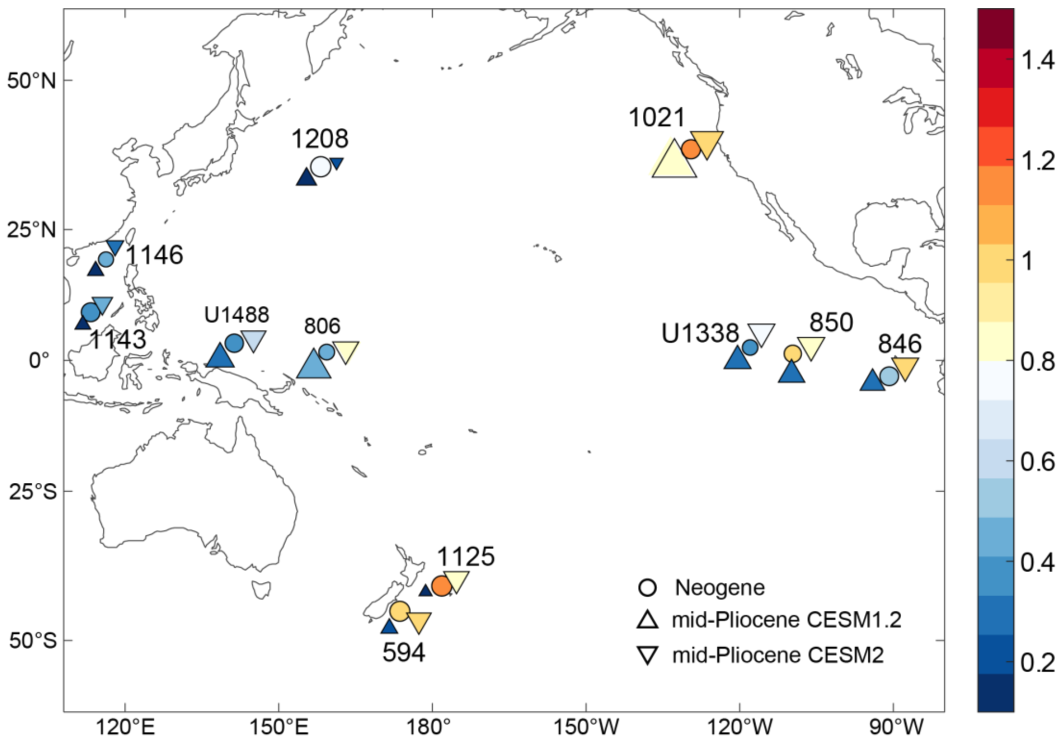

Supplementary Fig. 13. Single-site SST change relative to high-latitude averaged SST change over the past 10 Myr and the 1200-yr time series of the mid-Pliocene CESM simulations. Colors indicate the slope of the linear regression between the site-specific sea surface temperatures (SSTs) and high-latitude averaged SSTs. Circles represent the reconstructed data between 9.8 and 0.5 Ma except for Site 1146 which shows the relative SST change between 9.8 and 5 Ma, and upward-pointing and downward-pointing triangles represent the mid-Pliocene model outputs generated by the Community Earth System Model versions 1.2 (CESM1.2) and 2 (CESM2), respectively.

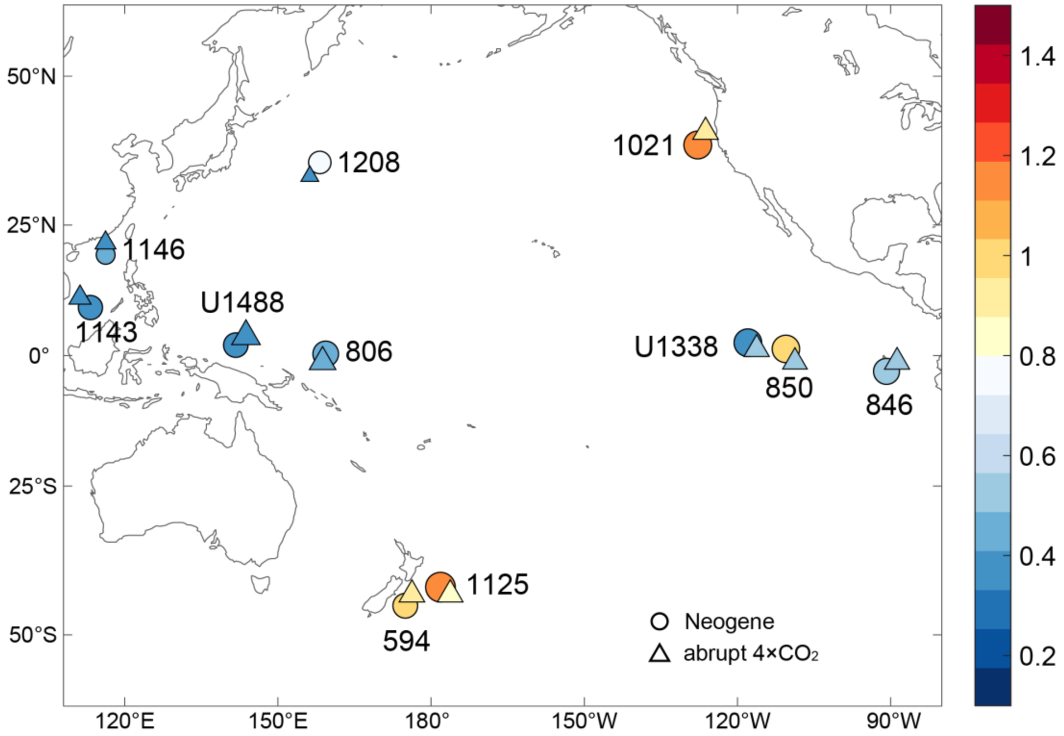

Supplementary Fig. 14. Single-site SST change relative to high-latitude averaged SST change over the past 10 Myr and the whole time series of the CESM104 simulation. Colors indicate the slope of the linear regression between the site-specific sea surface temperatures (SSTs) and high-latitude averaged SSTs. Circles represent the reconstructed data between 9.8 and 0.5 Ma except for Site 1146 which shows the relative SST change between 9.8 and 5 Ma, and triangles represent the model outputs generated by the Community Earth System Model version 1.0.4 (CESM104) simulation with abrupt quadrupled-CO_2 (4×CO_2) forcing.

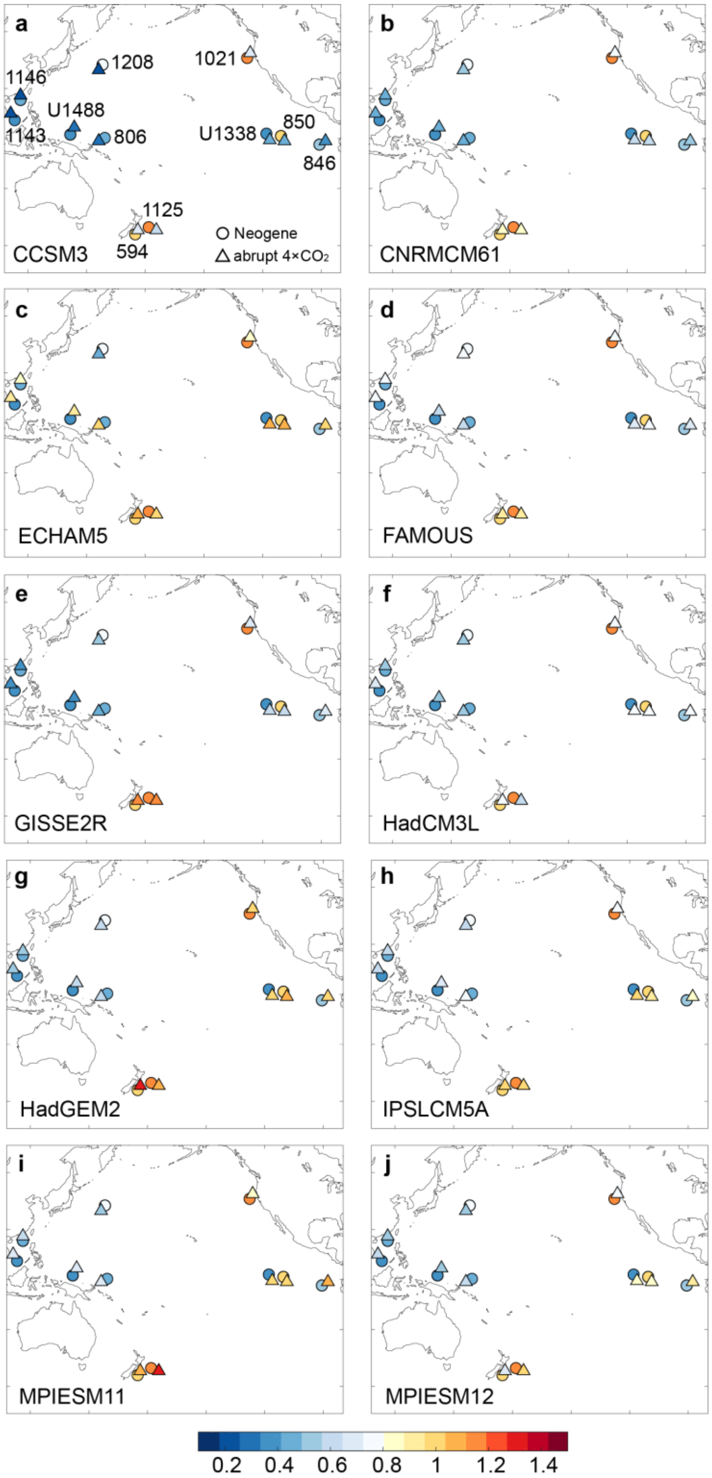

Supplementary Fig. 15. Comparison between the Neogene data and model outputs on single-site sea surface temperature change relative to high-latitude averaged sea surface temperature change. **a**, **b**, **c**, **d**, **e**, **f**, **g**, **h**, **i**, and **j** show the comparison between the Neogene data

and model-derived results from CCSM3, CNRMCM61, ECHAM5, FAMOUS, GISSE2R, HadCM3L, HadGEM2, IPSLCM5A, MPIESM11, and MPIESM12, respectively. Circles represent the reconstructed temperature data and triangles represent model outputs with abrupt quadrupled-CO_2 (4×CO_2) forcing. Colors indicate the slope of the linear regression between the site-specific SSTs and high-latitude averaged SSTs.

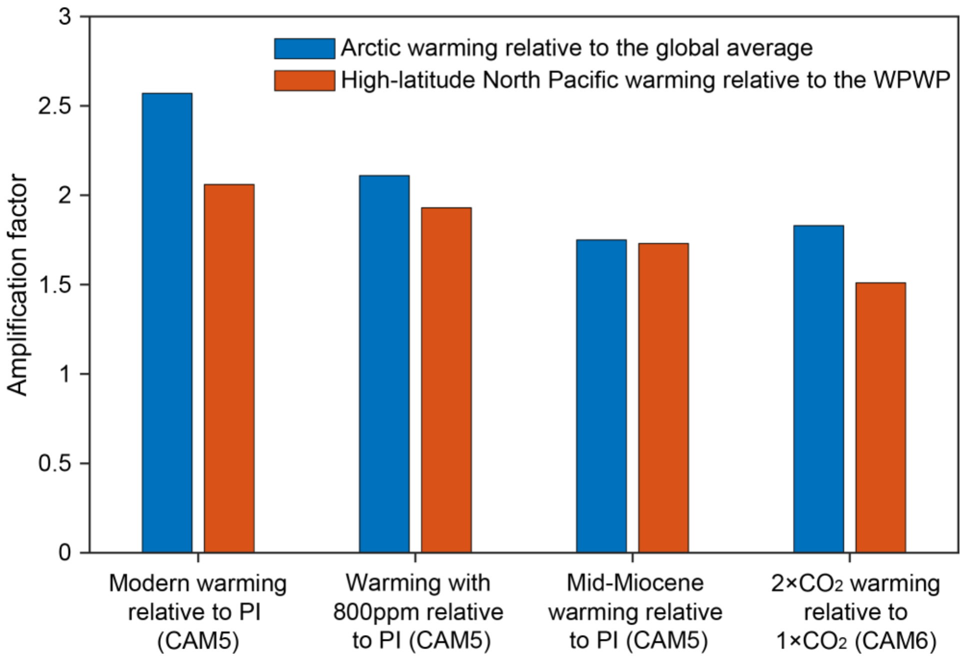

Supplementary Fig. 16. Comparison between Arctic amplification factor and Pacific high latitude amplification factor defined in our study. High latitude amplification factor is defined as the normalized high-latitude temperature change by the temperature change of the Western Pacific Warm Pool (WPWP). The amplification factors are estimated from the temperature outputs generated by the Community Earth System Model simulations with the Community Atmosphere Model version 5 (CAM5) or 6 (CAM6).

Supplementary Table 1. Calibrating the biostratigraphy of Site 806 to the Geological Time Scale 2012. LO is short for last occurrence and FO is short for first occurrence. The depth of biostratigraphic events at Site 806B were obtained from Nathan and Leckie[19].

Biostratigraphic event	806B (mbsf)	Datum Age (Ma)
LO *T. rugosus*	151.38	5.28
LO *D. quinqueramus*	160.88	5.59
FO *A. amplificus*	213.13	6.91
FO *D. berggrenii*	284.38	8.29
FO *R. pseudoumbilicus* paracme	310.56	8.79
LO *D. hamatus*	336.93	9.53
FO *D. hamatus*	365.28	10.55
FO *C. coalitus*	374.98	10.89
LO *G. fohsi* (foraminifer)	412.79	11.79
LO *S. heteromorphus*	475.68	13.53

Supplementary Table 2. Pacific site information. All Western Pacific Warm Pool (WPWP) sites are within the 28.5°C isotherm that is used to define the modern warm pool. The middle latitudes are defined as from 30° to 50°. The high latitudes sites are from >50°N, with no available site from the high-latitude South Pacific during our studied interval.

Region	Sites	SST-proxy data and time periods (Ma)	References
WPWP	806	TEX_{86}, 10.14-0.1<br>Mg/Ca, 5.14-0.02; Mg/Ca, 12.49-5.77	[13]<br>[20]; this study
	U1488	TEX_{86} and $U^{K'}_{37}$, 9.75-0.01	This study
	1143	TEX_{86}, 4.99-0.05; TEX_{86}, 10.69-2.78; $U^{K'}_{37}$, 4-0	[21]; [13]; [22]
Eastern Equatorial Pacific	U1338	$U^{K'}_{37}$, 16.04-0.42	[14,15]
	850	TEX_{86} and $U^{K'}_{37}$, 11.88-0	[13]
	846	$U^{K'}_{37}$, 12.34-0	[6]
Middle latitudes	1208	$U^{K'}_{37}$, 9.78-0.01	[6]
	1021	$U^{K'}_{37}$, 13.11-0.04	[6]
	594	$U^{K'}_{37}$, 12.36-2.74	[6]
	1125	$U^{K'}_{37}$, 11.12-0	[6]
High latitudes	882	$U^{K'}_{37}$, 3.64-0; $U^{K'}_{37}$, 5.87-0.48	[16]; [17]
	883/884	$U^{K'}_{37}$, 11.35-2.71	[6]
	887	$U^{K'}_{37}$, 7.33-5.15	[6]

Supplementary Table 3. Information of models with abrupt 4×CO_2 forcing and statistical analyses of the linear regression for the whole time series shown in Fig. 6. Estimated equilibrium warming for doubling CO_2 are obtained from Rugenstein et al[23] and the ordinary least squares regression was used to calculate the slope and standard error (SE). WPWP is short for Western Pacific Warm Pool.

Model name	Estimated equilibrium warming for doubling CO_2	WPWP *vs.* high latitudes		Meridional gradients *vs.* high latitudes		Middle *vs.* high latitudes	
		slope	SE	slope	SE	slope	SE
CCSM3	2.73	0.29	0.008	-0.71	0.01	0.54	0.017
CESM104	3.38	0.43	0.009	-0.57	0.01	0.75	0.016
CNRMCM61	5.7	0.47	0.017	-0.53	0.02	0.72	0.016
ECHAM5	5.83	0.98	0.022	-0.02	0.02	0.82	0.029
FAMOUS	8.55	0.58	0.007	-0.42	0.01	0.78	0.019
GISSE2R	2.44	0.42	0.016	-0.58	0.02	0.82	0.022
HadCM3L	3.45	0.53	0.021	-0.47	0.02	0.64	0.024
HadGEM2	4.77	0.61	0.017	-0.39	0.02	0.94	0.024
IPSLCM5A	4.76	0.73	0.019	-0.27	0.02	0.85	0.036
MPIESM11	3.42	0.65	0.020	-0.35	0.02	0.85	0.027
MPIESM12	3.35	0.52	0.022	-0.48	0.02	0.72	0.023

Supplementary Table 4. Pacific high latitude amplification and Arctic amplification factor estimated from zonally averaged surface air temperature anomaly over the last 30 years of each simulation with abrupt 4×CO_2 forcing. The surface air temperature (tas) anomaly is the temperature difference of the experiment relative to the mean of the pre-industrial control simulation. High latitude amplification factor is the ratio of high latitude tas anomaly relative to the Western Pacific Warm Pool (WPWP), and Arctic amplification factor is the ratio of Arctic (67.5°N to 90°N) tas anomaly relative to the global mean.

Model long name<br>Short name	Average tas anomaly over the last 30 yr of each simulation (°C)				High latitude amplification factor	Arctic amplification factor
	High latitude	WPWP	Polar (60°N-90°N)	Global		
CCSM3<br>CCSM3	6.89 ± 0.44	3.27 ± 0.11	10.17 ± 0.49	6.14 ± 0.11	2.11	1.66
CESM1.0.4<br>CESM104	8.30 ± 0.38	4.25 ± 0.22	16.05 ± 0.39	8.55 ± 0.11	1.95	1.88
CNRM-CM6-1<br>CNRMCM61	9.64 ± 0.38	6.67 ± 0.14	20.10 ± 0.36	11.20 ± 0.09	1.45	1.80
ECHAM5/MPI OM<br>ECHAM5	10.19 ± 0.22	10.36 ± 0.36	17.43 ± 0.36	11.67 ± 0.22	0.98	1.49
FAMOUS<br>FAMOUS	22.35 ± 0.44	10.62 ± 0.21	28.52 ± 0.27	16.31 ± 0.12	2.10	1.75
GISS-E2-R<br>GISSE2R	4.67 ± 0.37	3.19 ± 0.16	8.23 ± 0.41	5.56 ± 0.08	1.46	1.48
HadCM3L<br>HadCM3L	7.52 ± 0.26	4.50 ± 0.16	10.28 ± 0.27	6.57 ± 0.15	1.67	1.57
HadGEM2-ES<br>HadGEM2	10.33 ± 0.33	5.67 ± 0.12	19.77 ± 0.29	10.33 ± 0.13	1.82	1.91
IPSL-CM5A-LR<br>IPSLCM5A	7.23 ± 0.30	6.40 ± 0.16	12.86 ± 0.37	8.00 ± 0.11	1.13	1.61
MPI-ESM 1.1<br>MPIESM11	7.87 ± 0.28	5.39 ± 0.15	14.95 ± 0.30	8.33 ± 0.13	1.46	1.79
MPI-ESM 1.2<br>MPIESM12	7.50 ± 0.28	5.07 ± 0.13	14.24 ± 0.21	7.56 ± 0.09	1.48	1.88

Supplementary References

1. Conte, M. H. *et al.* Global temperature calibration of the alkenone unsaturation index ($U^{K'}_{37}$) in surface waters and comparison with surface sediments. *Geochemistry, Geophysics, Geosystems* **7**, Q02005 (2006).
2. Hollstein, M. *et al.* Stable oxygen isotopes and Mg/Ca in planktic foraminifera from modern surface sediments of the Western Pacific Warm Pool: Implications for thermocline reconstructions. *Paleoceanography* **32**, 1174–1194 (2017).
3. Tierney, J. E., Malevich, S. B., Gray, W., Vetter, L. & Thirumalai, K. Bayesian calibration of the Mg/Ca paleothermometer in planktic foraminifera. *Paleoceanography and Paleoclimatology* (2019).
4. Gray, W. R. & Evans, D. Nonthermal influences on Mg/Ca in planktonic foraminifera: A review of culture studies and application to the last glacial maximum. *Paleoceanography and Paleoclimatology* **34**, 306–315 (2019).
5. Dekens, P. S., Lea, D. W., Pak, D. K. & Spero, H. J. Core top calibration of Mg/Ca in tropical foraminifera: Refining paleotemperature estimation. *Geochemistry, Geophysics, Geosystems* **3**, 1–29 (2002).
6. Herbert, T. D. *et al.* Late Miocene global cooling and the rise of modern ecosystems. *Nature Geoscience* **9**, 843 (2016).
7. Holbourn, A. E. *et al.* Late Miocene climate cooling and intensification of southeast Asian winter monsoon. *Nature communications* **9**, 1584 (2018).
8. Stanley, S. M. & Hardie, L. A. Secular oscillations in the carbonate mineralogy of reef-building and sediment-producing organisms driven by tectonically forced shifts in seawater chemistry. *Palaeogeography, Palaeoclimatology, Palaeoecology* **144**, 3–19 (1998).
9. Rugenstein, M. *et al.* LongRunMIP: Motivation and Design for a Large Collection of Millennial-Length AOGCM Simulations. *Bulletin of the American Meteorological Society* **100**, 2551–2570 (2020).
10. Collins, M. *et al.* Long-term Climate Change: Projections, Commitments and Irreversibility. in *Climate Change 2013: The Physical Science Basis. Contribution of Working Group I to the Fifth Assessment Report of the Intergovernmental Panel on Climate Change* (eds. Stocker, T. F. et al.) 1029–1136 (Cambridge University Press, 2013). doi:10.1017/CBO9781107415324.024.
11. Evans, D., Brierley, C., Raymo, M. E., Erez, J. & Müller, W. Planktic foraminifera shell chemistry response to seawater chemistry: Pliocene–Pleistocene seawater Mg/Ca, temperature and sea level change. *Earth and Planetary Science Letters* **438**, 139–148 (2016).
12. Sosdian, S. M. & Lear, C. H. Initiation of the Western Pacific Warm Pool at the Middle Miocene Climate Transition? *Paleoceanography and Paleoclimatology* **35**, e2020PA003920 (2020).

13. Zhang, Y. G., Pagani, M. & Liu, Z. A 12-million-year temperature history of the tropical Pacific Ocean. *Science* **344**, 84–87 (2014).
14. Rousselle, G., Beltran, C., Sicre, M.-A., Raffi, I. & De Rafélis, M. Changes in sea-surface conditions in the Equatorial Pacific during the middle Miocene–Pliocene as inferred from coccolith geochemistry. *Earth and Planetary Science Letters* **361**, 412–421 (2013).
15. Beltran, C. *et al.* Evolution of the Zonal Gradients Across the Equatorial Pacific During the Miocene–Pleistocene. *Journal of Sedimentary Research* **89**, 242–252 (2019).
16. Martínez-Garcia, A., Rosell-Melé, A., McClymont, E. L., Gersonde, R. & Haug, G. H. Subpolar link to the emergence of the modern equatorial Pacific cold tongue. *Science* **328**, 1550–1553 (2010).
17. Yamamoto, M. & Kobayashi, D. Surface ocean cooling in the subarctic North Pacific during the late Pliocene suggests an atmospheric reorganization prior to extensive Northern Hemisphere glaciation. *Deep Sea Research Part II: Topical Studies in Oceanography* **125**, 177–183 (2016).
18. Tierney, J. E. & Tingley, M. P. BAYSPLINE: a new calibration for the alkenone paleothermometer. *Paleoceanography and Paleoclimatology* **33**, 281–301 (2018).
19. Nathan, S. A. & Leckie, R. M. Early history of the Western Pacific Warm Pool during the middle to late Miocene (~13.2–5.8 Ma): Role of sea-level change and implications for equatorial circulation. *Palaeogeography, Palaeoclimatology, Palaeoecology* **274**, 140–159 (2009).
20. Wara, M. W., Ravelo, A. C. & Delaney, M. L. Permanent El Niño-like conditions during the Pliocene warm period. *Science* **309**, 758–761 (2005).
21. O'Brien, C. L. *et al.* High sea surface temperatures in tropical warm pools during the Pliocene. *Nature Geoscience* **7**, 606–611 (2014).
22. Li, L. *et al.* A 4-Ma record of thermal evolution in the tropical western Pacific and its implications on climate change. *Earth and Planetary Science Letters* **309**, 10–20 (2011).
23. Rugenstein, M. *et al.* Equilibrium Climate Sensitivity Estimated by Equilibrating Climate Models. *Geophysical Research Letters* **47**, e2019GL083898 (2020).
